# Metal- and solvent-free synthesis of amides using substitute formamides as an amino source under mild conditions

**DOI:** 10.1038/s41598-019-39240-z

**Published:** 2019-02-26

**Authors:** Feng Zhang, Lesong Li, Jingyu Zhang, Hang Gong

**Affiliations:** 1 College of Science, Hunan Agricultural University, Changsha, 410128 China; 2 The Key Laboratory of Environmentally Friendly Chemistry and Application of the inistry of Education, The Key Laboratory for Green Organic Synthesis and Application of Hunan Province, College of Chemistry, Xiangtan University, Xiangtan, 411105 China

## Abstract

This study described an efficient and practical approach for amide synthesis. The reaction was conducted under metal- and solvent-free conditions at a mild temperature (40 °C) in air, and readily available formamides were used as an amino source. This reaction can be easily upgraded to the gram level with an excellent yield.

## Introduction

**Figure 1:**
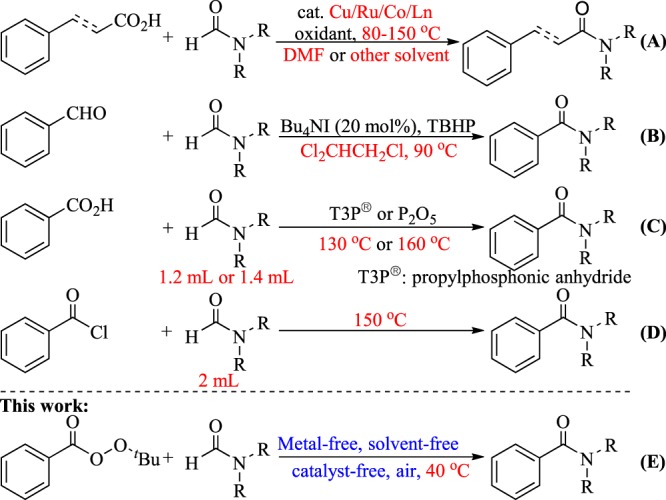
Cross-coupling reaction of formamide with a carboxylic acid derivative.

Amide is one of the primary components of biomolecules, such as proteins, and is also commonly found in natural products, pharmaceuticals, pesticides, and functional materials^1–3^. Amide synthesis has attracted continuous interest, and various methods have been developed^4–7^. Formamides are cheap, readily available, and versatile organic compounds that are commonly used as solvents and as a source for carbonyl, dimethylamino, and Me_2_NCO^8–12^. Based on the application potential and environmental benign aspects, the coupling reaction of formamide with carboxylic acid derivative shows promise as a synthesis method for amides. However, these coupling strategies are often conducted in the presence of a metal catalyst, such as Cu^13–20^, Ru^21^, Co^22^, and Ln^23^, thereby resulting to the production of metal residues and the excessive use of DMF as a solvent (Fig. 1A). Additionally, high reaction temperature of 80 °C to 150 °C is required. Several metal-free cross-coupling reactions of formamide with a carboxylic acid derivative were also developed. Recently, Wan *et al*. reported Bu_4_NI-catalyzed cross-coupling of formamide with aldehyde using TBHP as the oxidant and Cl_2_CHCH_2_Cl as the solvent; in their study, 25 equiv of DMF and a high reaction temperature of 90 °C are still required (Fig. 1B)^24^. Wolf also reported a metal-free oxidative amination of aldehydes to amides using TBHP as oxidant^25^. Mavel and Tortoioli reported that phosphorus-containing compounds promote the coupling reaction of formamide with carboxylic acid (Fig. 1C)^26,27^. However, the use of a large amount of phosphorus reagent, the high reaction temperature above 130 °C, and the excessive wastage of formamides are not environmentally friendly. Yoon *et al*. reported the coupling reaction of acid chloride with DMF, which also required a high reaction temperature and resulted to the wastage of DMF (Fig. 1D)^28^. In this study, we synthesized valuable amides by a metal- and solvent-free method conducted at 40 °C in air under mild conditions (Fig. 1E).

## Results and Discussion

**Figure 2:**
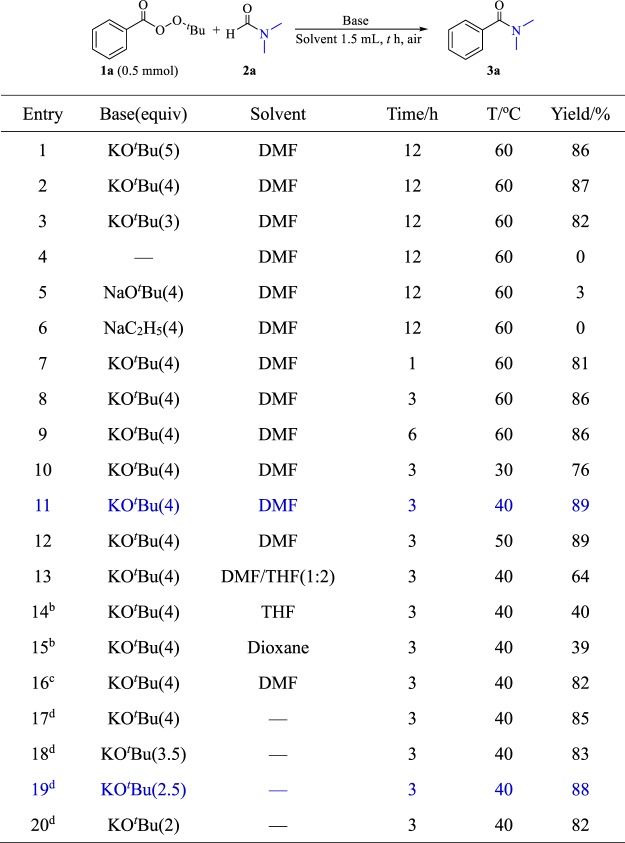
Selected optimization results^a^. ^a^Unless otherwise noted, all reactions were conducted on a 0.5 mmol scale; Yields were determined by ^1^H NMR spectroscopy using nitromethane as internal standard. ^b^Using DMF (5 equiv, 194 μL). ^c^Under the atmosphere of argon. ^d^Solvent free, using DMF (5 equiv, 194 μL).

In the initial study, the reaction of *tert*-butyl benzoperoxoate (0.5 mmol) and DMF (1.5 mL) was investigated in the presence of KO^*t*^Bu (Fig. 2, entry 1). The desired product was obtained in an 86% yield. A series of optimization reactions, including the optimization of the amount and type of base (Fig. 2, entries 1–6; Table S1, entries 1–14), the reaction time (Fig. 2, entries 7–9; Table S1, entries 15–18), the reaction temperature (Fig. 2, entries 10–12; Table S1, entries 19–22), the solvent (Fig. 2, entries 13–15; Table S1, entries 23–26), and the reaction atmosphere (Fig. 2, entry 16; Table S1, entry 27) was conducted. The reaction was also conducted under solvent-free conditions and yielded almost the same amount (85%) (Fig. 2, entry 17; Table S1, entry 28). A good yield of 88% was achieved after adjusting the amount of KO^*t*^Bu to 2.5 equiv (Fig. 2, entry 19; Table S1, entry 30).

**Figure 3:**
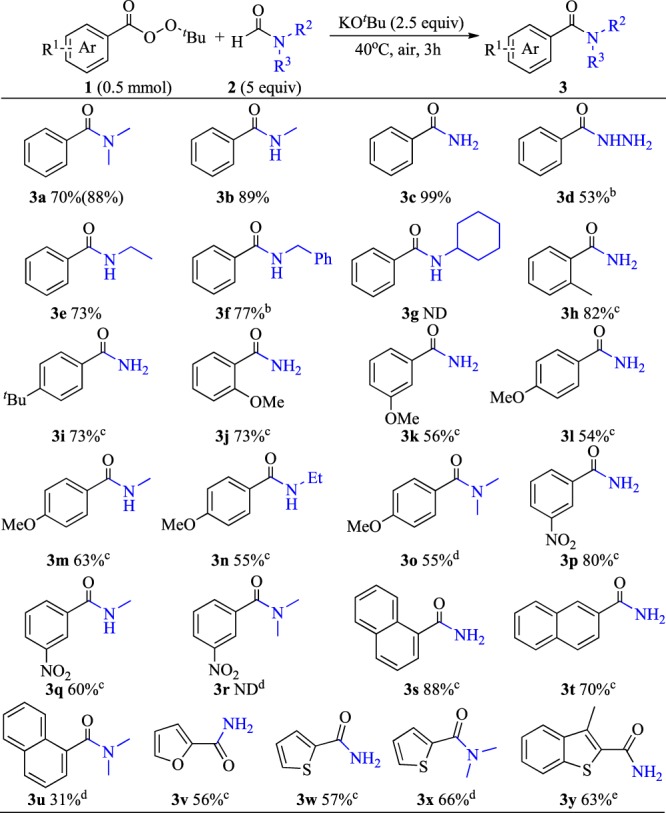
Scope of amide synthesis^a^. ^a^Unless otherwise noted, all reactions were conducted on a 0.5 mmol scale, amide compounds (5 equiv), KO^*t*^Bu (2.5 equiv, 140 mg) in a sealed tube under an atmosphere of air for 3 h. Isolated yield was showed out brackets, ^1^H NMR yield were showed in brackets. ^b^10 equiv of amide 2 was used, temperature is 70 °C. ^c^10 equiv of amide 2 was used, temperature 80 °C. ^d^1.5 mL DMF was used, 4 equiv KO^*t*^Bu was used, temperature is 80 °C. ^e^Peroxide (0.1 mmol), KO^*t*^Bu (5 equiv), HCONH_2_ (25 equiv), temperature is 80 °C.

The generalizability of this method was explored under the following optimized conditions: peroxoate, 0.5 mmol; substrate amide, 5 equiv; and KO^*t*^Bu, 4 equiv at 40 °C in air for 3 h. *N,N*-disubstituted, *N*-monosubstituted, and unsubstituted amides **2** were converted into the desired product **3** with good to excellent yields (Fig. 3a–c). In addition, formyl hydrazine smoothly reacted with *tert*-butylbenzoperoxoate and obtained the corresponding product with a moderate yield (**3d**). When the hydrophobicity of the alkyl substituents on the nitrogen of amide **2** was increased, the solubility of KO^*t*^Bu decreased, which is unfavorable to this conversion (**3e**–**f**). No product was detected when *N*-cyclohexyl formamide was used as the amino source (**3 g**). Other *tert*-butylbenzoperoxoate derivatives were also investigated and found that electron-rich or electron-deficient derivatives render this reaction smooth (**3h**–**q**). Naphthylperoxoates also showed high reactivity and obtained good isolated yields (**3s**–**t**). Heteroaromatic (including furan, thiophene, and benzothiophene) peroxoates can also be converted into corresponding products with moderate yields (**3v**–**y**). However, **3r** and **3 u** were either undetected or with only a poor yield, which might be caused by the aforementioned poor solubility of KO^*t*^Bu on the reaction systems. The reaction of alkyl perester, such as *tert*-butyl ethaneperoxoate with *N*-benzylformamide was conducted, but no amide product could be detected.

**Figure 4:**
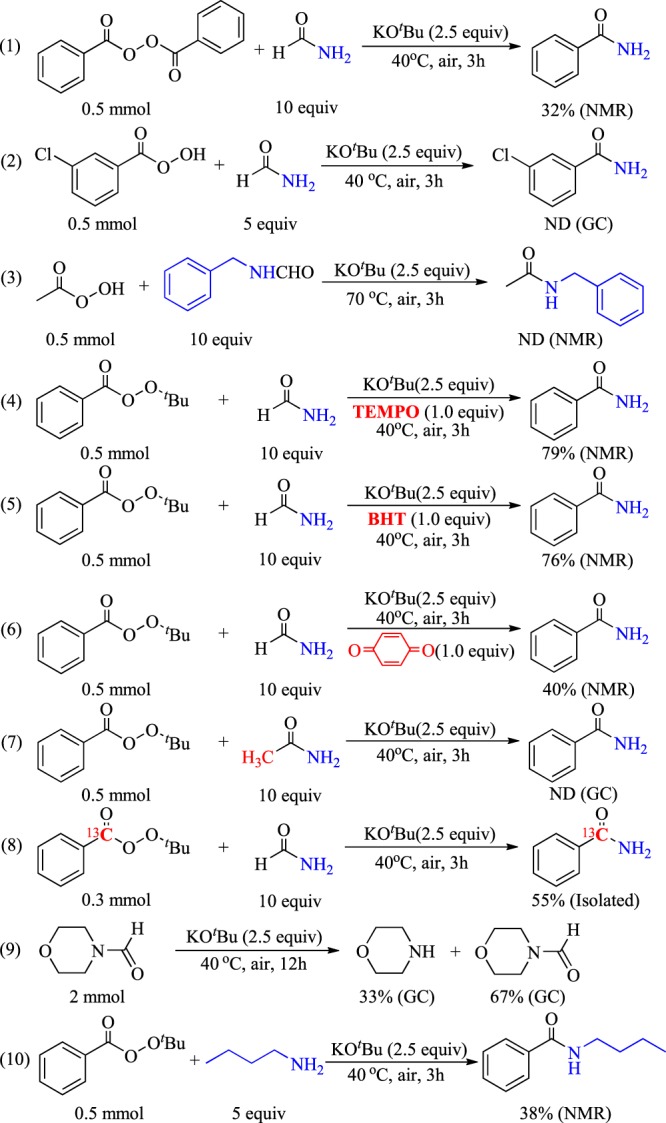
Control experiments.

Several control experiments were conducted to investigate the reaction mechanism. First, we used benzoyl peroxide instead of *tert*-butylbenzoperoxoate and obtained a poor yield of 32% (Fig. 4, eq. 1). When one of the benzene rings is converted into the product, the remaining ring would probably lose its reactivity. The reaction of other peroxide acids such as 3-chloroperoxybenzoic acid and peroxyacetic acid with formamide derivatives were also tried, but no desired product could be detected (Fig. 4, eqs 2 and 3).

**Figure 5:**
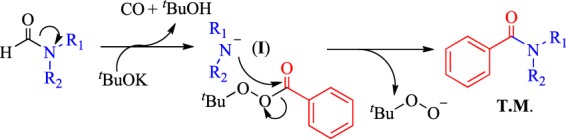
Proposed mechanism.

From a general perspective, this transformation was considered as radical process because peroxide was used as a substrate. Hence, the radical block reaction was conducted using TEMPO as a radical inhibitor. The addition of TEMPO showed no evident effect, and the product yield was 79% (Fig. 4, eq. 4). Good and moderate yields were still obtained when BHT and benzoquinone were used as inhibitors, respectively (Fig. 4, eqs 5 and 6). These results excluded the radical process of this transformation. Afterwards, the hydrogen of aldehyde was replaced by methyl, and almost no desired product was detected (Fig. 4, eq. 7). This result indicated the need for decarbonylation, which was blocked by methyl. Then, an isotope labeling experiment was conducted to confirm the source of carbonyl on the target molecules (**T.M**.) (Fig. 4, eq. 8). Almost all carbon molecules in the carbonyl group of the product were identified as isotope ^13^C. Afterward, the decomposition reaction of formamide derivative was tested, and it was found morpholine-4-carbaldehyde could be decomposed to morpholine under the standard condition (Fig. 4, eq. 9). Followed, the reaction of *tert*-butyl benzoperoxoate and amine were conducted, and the desired product was found with a yield of 38% (Fig. 4, eq. 10). These results indicated that the decomposition of formamide derivative and the corresponding decomposition product amine might be played an important role in this amide synthesis procedure. Based on these control experiments and previous studies^13,29^, a possible mechanism was proposed (Fig. 5).

Initially, dimethylamine anion (**I**) was formed via the decarbonylation of formamide with the release of CO in the presence of KO^*t*^Bu^30^. The nucleophilic addition of (**I**) to *tert*-butylbenzoperoxoate subsequently occurred while gathering the **T.M**.

**Figure 6:**
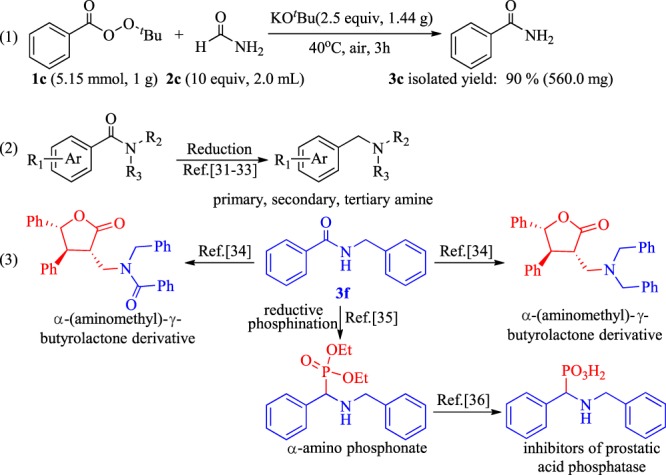
Gram-scale reaction and application of amides.

A gram-scale reaction of **1c** was conducted to verify its potential in industrial production, and an excellent isolated yield of 90% was achieved (Fig. 6, eq. 1). Amides are known to have great potential application. For example, amides can be easily converted into amines (including primary, secondary, and tertiary amines) through reduction reactions (Fig. 6, eq. 2)^31–33^. Another example is that product **3f** can be converted into bioactive molecules (Fig. 6, eq. 3)^34–36^.

## Conclusions

In summary, an efficient and practical approach for the synthesis of amide has been developed. The reaction is conducted in air at a mild temperature (40 °C) under metal- and solvent-free conditions, and the readily available substitute formamides were used as an amino source. This transformation can easily be upgraded to the gram level, thereby providing an avenue for the synthesis of valuable amides.

## Materials and Methods

### General information

Preparative thin-layer chromatography was performed for product purification using Sorbent Silica Gel 60 F254 TLC plates and visualized with ultraviolet light. IR spectra were recorded on a new Fourier transform infrared spectroscopy. ^1^H, ^13^C and ^19^F NMR spectra were recorded on 400, 100, 377 MHz NMR spectrometer using CDCl_3_ as solvent unless otherwise stated. HRMS were made by means of ESI. Melting points were measured on micro melting point apparatus and uncorrected. Unless otherwise noted, all reagents were weighed and handled in air, and all reactions were carried out in a sealed tube under an atmosphere of air. Unless otherwise noted, all reagents were purchased from reagent company, and used without further purifications.

### Experimental Section

A typical experimental procedure for transamidation was conducted as follows: A solution of peroxoate (0.5 mmol), KO^*t*^Bu (2.5 equiv, 140 mg) and amide (5 equiv or 10 equiv) were stirred in a sealed tube under an atmosphere of air at 40 °C for 3 h. The reaction mixture was then extracted with ethyl acetate. Afterward, the solution was evaporated under vacuum. The residue was purified by preparative thin-layer chromatography (TLC) on silica gel with petroleum ether and ethyl acetate (5% triethylamine) to achieve the pure product.

## Supplementary information

Supplementary Information (traceless)
