# Supplementary material for: Metal- and solvent-free synthesis of amides using substitute formamides as an amino source under mild conditions
Source: Sci Rep. 2019 Feb 26;9:2787. doi: 10.1038/s41598-019-39240-z (PMC6391388; doi:10.1038/s41598-019-39240-z)

| Metal-, Solvent- and Catalyst-free Synthesis of Amides Using Substitute Formamides as an Amino Source under Mild Conditions  Feng Zhang,^1,^* Lesong Li,^2^ Jingyu Zhang,^2^ Hang Gong*^,2^  ^1^College of Science, Hunan Agricultural University, Changsha, 410128, (China)  ^2^The Key Laboratory of Environmentally Friendly Chemistry and Application of the Ministry of Education; The Key Laboratory for Green Organic Synthesis and Application of Hunan Province, College of Chemistry, College of Chemistry, Xiangtan University, Xiangtan 411105, China.  (E-mail: zhangf@iccas.ac.cn; E-mail: hgong@xtu.edu.cn)  **Table of Contents**   1. General information...............................................................................................S1 2. Selected optimization results.................................................................................S1 3. Synthesis and characterization of substrates.........................................................S2 4. Experimental procedure and characterization data for products...........................S5 5. Gram-scale synthesis of Benzamide (3c) .............................................................S10 6. References.............................................................................................................S11 7. Copies of ^1^H and ^13^C NMR spectra .....................................................................S12 |
| --- |

1. **General information**

Preparative thin-layer chromatography was performed for product purification using Sorbent Silica Gel 60 F254 TLC plates and visualized with ultraviolet light. IR spectra were recorded on a new Fourier transform infrared spectroscopy.^1^H, ^13^C and ^19^F NMR spectra were recorded on 400, 100, 377 MHz NMR spectrometer using CDCl3 as solvent unless otherwise stated. HRMS were made by means of ESI. Melting points were measured on micro melting point apparatus and uncorrected. Unless otherwise noted, all reagents were weighed and handled in air, and all reactions were carried out in a sealed tube under an atmosphere of air. Unless otherwise noted, all reagents were purchased on the net, and used without further purifications.

1. **Selected optimization results**

**Table S1**. Selected Optimization Results ^a^

| Entry | Base(equiv) | Solvent(1.5 mL) | Time(h) | T(^o^C) | | Yeild/% ^b^ |
| --- | --- | --- | --- | --- | --- | --- |
| 1 | KO*^t^*Bu(5) | DMF | 12 | 60 | 86 | |
| 2 | KO*^t^*Bu(4) | DMF | 12 | 60 | 87 | |
| 3 | KO*^t^*Bu(3) | DMF | 12 | 60 | 82 | |
| 4 | KO*^t^*Bu(2) | DMF | 12 | 60 | 54 | |
| 5 | KO*^t^*Bu(1) | DMF | 12 | 60 | 21 | |
| 6 | KO*^t^*Bu(0) | DMF | 12 | 60 | 0 | |
| 7 | NaO*^t^*Bu(4) | DMF | 12 | 60 | 3 | |
| 8 | NaC_2_H_5_(4) | DMF | 12 | 60 | 0 | |
| 9 | CH_3_COONa(4) | DMF | 12 | 60 | 0 | |
| 10 | CH_3_COOK(4) | DMF | 12 | 60 | 0 | |
| 11 | NaOH(4) | DMF | 12 | 60 | 0 | |
| 12 | KOH(4) | DMF | 12 | 60 | 0 | |
| 13 | Na_2_CO_3_(4) | DMF | 12 | 60 | 0 | |
| 14 | K_2_CO_3_(4) | DMF | 12 | 60 | 0 | |
| 15 | KO*^t^*Bu(4) | DMF | 1 | 60 | 81 | |
| 16 | KO*^t^*Bu(4) | DMF | 3 | 60 | 86 | |
| 17 | KO*^t^*Bu(4) | DMF | 6 | 60 | 86 | |
| 18 | KO*^t^*Bu(4) | DMF | 18 | 60 | 71 | |
| 19 | KO*^t^*Bu(4) | DMF | 3 | 30 | 76 | |
| 20 | KO*^t^*Bu(4) | DMF | 3 | 40 | 89 | |
| 21 | KO*^t^*Bu(4) | DMF | 3 | 50 | 89 | |
| 22 | KO*^t^*Bu(4) | DMF | 3 | 80 | 79 | |
| 23 | KO*^t^*Bu(4) | DMF/THF(1:2) | 3 | 40 | 64 | |
| 24 | KO*^t^*Bu(4) | DMF/Diox(1:2) | 3 | 40 | 58 | |
| 25^c^ | KO*^t^*Bu(4) | THF | 3 | 40 | 40 | |
| 26^c^ | KO*^t^*Bu(4) | Dioxane | 3 | 40 | 39 | |
| 27^d^ | KO*^t^*Bu(4) | DMF | 3 | 40 | 82 | |
| 28^e^ | KO*^t^*Bu(4) | — | 3 | 40 | 85 | |
| 29^e^ | KO*^t^*Bu(3.5) | — | 3 | 40 | 83 | |
| 30^e^ | KO*^t^*Bu(2.5) | — | 3 | 40 | 88 | |
| 31^e^ | KO*^t^*Bu(2) | — | 3 | 40 | 82 | |
| 32^e^ | KO*^t^*Bu(1.5) | — | 3 | 40 | 80 | |
| 33^e^ | KO*^t^*Bu(1) | — | 3 | 40 | 54 | |

**^a^** Unless otherwise noted, all reactions were conducted on a 0.5 mmol scale; **^b^** Yields were determined by ^1^H NMR spectroscopy using nitromethane as internal standard; **^c^** using DMF(5 equiv, 194 μL). **^d^** under the atmosphere of argon; ^e^ solvent free, using DMF(5 equiv, 194 μL).

**3. Synthesis and characterization of substrates**

**The synthesis of compound** **1l**

*tert*-Butyl 4-methoxybenzoperoxoate (**1l**) were prepared using the reported strategy^[1]^: A solution of 4-methoxybenzyl alcohol (20 mmol), Bu_4_NI (20 mol%) and TBHP(*tert*-butyl hydroperoxide, 70% solution in water) (0.1 mol) in H_2_O (70 mL) stirred 24 h at 40 ^o^C. Afterward, the solution was extracted with ethyl acetate (3×20 mL), the combined organic layers were dried with over anhydrous Na_2_SO_4_. The solvent was evaporated under vacuum and the crude product was purified by silica gel column chromatography to give compound **1l** (isolate yield 88%).

*tert*-Butyl 4-methoxybenzoperoxoate (**1l)**: ^1^H NMR (400 MHz, CDCl_3_) δ 7.92 (d, *J* = 8.8 Hz, 2H), 6.94 (d, *J* = 8.8 Hz, 2H), 3.87 (s, 3H), 1.41 (s, 9H); ^13^C NMR (100 MHz, CDCl_3_) δ 164.29 (s), 163.64 (s), 131.22 (s), 119.84 (s), 113.93 (s), 83.82 (s), 55.50 (s), 26.28 (s).

**The synthesis of compound 1h-k**, **1p**, **1s-t**, **1v-w**.

Compound **1h-k**, **1p**, **1s-t**, **1v-w**. were prepared using the reported strategy^[2]^: A solution of Aromatic aldehydes (4–5 mmol), Bu_4_NI (30 mol%) and TBHP (*tert*-butyl hydroperoxide, 70% solution in water) (5 equiv) in H_2_O (9–15 mL) stirred 24 h at 50 ^o^C. Afterward, the solution was extracted with ethyl acetate (3×20 mL), the combined organic layers were dried with over anhydrous Na_2_SO_4_. The solvent was evaporated under vacuum and the crude product was purified by silica gel column chromatography to give the desired product .

*tert*-Butyl 2-methylbenzoperoxoate **(1h)**: isolate yield: 82%, ^1^H NMR (400 MHz, CDCl_3_) δ 7.74 (d, *J* = 7.6 Hz, 1H), 7.43 (td, *J*  = 7.4, 1.2 Hz, 1H), 7.29–7.24 (m, 2H), 2.57 (s, 3H), 1.42 (s, 9H); ^13^C NMR (100 MHz, CDCl_3_) δ 165.86 (s), 139.64 (s), 132.34 (s), 131.78 (s), 129.78 (s), 127.72 (s), 125.91 (s), 83.82 (s), 26.45 (s), 21.32 (s).

*tert*-Butyl 4-(*tert*-butyl)benzoperoxoate **(1i)**: isolate yield: 73%, ^1^H NMR (400 MHz, CDCl_3_) δ 7.90 (d, *J* = 8.8 Hz, 2H), 7.48 (d, *J* = 8.4 Hz, 2H), 1.42 (s, 9H), 1.34 (s, 9H); ^13^C NMR (100 MHz, CDCl_3_) δ 164.65 (s), 157.25 (s), 129.16 (s), 125.75 (s), 124.91 (s), 84.03 (s), 35.28 (s), 31.19 (s), 26.38 (s).

*tert*-Butyl 2-methoxybenzoperoxoate **(1j)**: isolate yield: 73%, ^1^H NMR (400 MHz, CDCl_3_) δ 7.70 (dd, *J* = 7.7, 1.7 Hz, 1H), 7.51–7.47 (m, 3H), 6.99 (t, *J* = 14.2, 8.0 Hz, 2H), 3.90 (s, 3H), 1.41(s, 1H); ^13^C NMR (100 MHz, CDCl_3_) δ 165.22 (s), 158.65 (s), 133.86 (s), 131.50 (s), 120.52 (s), 118.09 (s), 111.97 (s), 83.82 (s), 56.03 (s), 26.37 (s).

*tert*-Butyl 3-methoxybenzoperoxoate **(1k)**: isolate yield: 73%, ^1^H NMR (400 MHz, CDCl_3_) δ 7.54 (d, *J* = 7.6 Hz, 1H), 7.48 (s, 1H), 7.37 (t, *J* = 7.8 Hz, 1H), 7.13 (dd, *J* = 8.4, 2.4 Hz, 1H), 3.86 (s, 3H), 1.42 (s, 9H); ^13^C NMR (100 MHz, CDCl_3_) δ 164.49 (s), 159.79 (s), 129.81 (s), 129.02 (s), 121.48 (s), 119.79 (s), 114.05 (s), 84.18 (s), 55.62 (s), 26.38 (s).

*tert*-Butyl 3-nitrobenzoperoxoate **(1p)**: isolate yield: 53%, ^1^H NMR (400 MHz, CDCl_3_) δ 8.78 (t, *J* = 1.9 Hz, 1H), 8.47 (ddd, *J* = 8.2, 2.2, 1.0 Hz, 1H), 8.32–8.29 (m, 1H), 7.71 (t, *J* = 8.0 Hz, 1H), 1.45 (s, 9H); ^13^C NMR (100 MHz, CDCl_3_) δ 162.48 (s), 148.46 (s), 134.96 (s), 130.14 (s), 129.60 (s), 127.97 (s), 124.23 (s), 84.90 (s), 26.40 (s).

*tert*-Butyl naphthalene-1-carboperoxoate **(1s)**: isolate yield: 93%, ^1^H NMR (400 MHz, CDCl_3_) δ 8.63 (d, *J* = 8.8 Hz, 1H), 8.04 (d, *J* = 8.4 Hz, 1H), 7.98 (d, *J* = 7.2 Hz, 1H), 7.89 (d, *J* = 8.0 Hz, 1H), 7.63 (t, *J* = 7.6 Hz, 1H), 7.56 (t, *J* = 7.4 Hz, 1H), 7.50 (t, *J* = 7.8 Hz, 1H), 1.46 (s, 9H); ^13^C NMR (100 MHz, CDCl_3_) δ 165.72 (s), 133.91 (s), 133.54 (s), 131.09 (s), 129.10 (s), 128.70 (s), 128.14 (s), 126.73 (s), 125.53 (s), 125.32 (s), 124.57 (s), 26.50 (s).

*tert*-Butyl naphthalene-2-carboperoxoate **(1t)**: isolate yield: 78%, ^1^H NMR (400 MHz, CDCl_3_) δ 8.53 (s, 1H), 7.96 (d, *J* = 8.4 Hz, 2H), 7.91–7.88 (m, 2H), 7.64–7.55 (m, 2H), 1.47 (s, 9H); ^13^C NMR (100 MHz, CDCl_3_) δ 164.80 (s), 135.74 (s), 132.55 (s), 130.88 (s), 129.45 (s), 128.71 (s), 128.64 (s), 127.97 (s), 127.07 (s), 124.98 (s), 124.63 (s), 84.24 (s), 26.45 (s).

*tert*-Butyl furan-2-carboperoxoate **(1v)**: isolate yield: 35%, ^1^H NMR (400 MHz, CDCl_3_) δ 7.61 (dd, *J* = 1.7, 0.8 Hz, 1H), 7.20 (dd, *J* = 3.5, 0.8 Hz, 1H), 6.53 (dd, *J* = 3.5, 1.8 Hz, 1H), 1.39 (s, 9H); ^13^C NMR (100 MHz, CDCl_3_) δ 157.24 (s), 146.97 (s), 141.58 (s), 118.60 (s), 112.02 (s), 84.58 (s), 26.28 (s).

*tert*-Butyl thiophene-2-carboperoxoate **(1w)**: isolate yield: 90%, ^1^H NMR (400 MHz, CDCl_3_) δ 7.81 (d, *J* = 3.2 Hz, 1H), 7.61 (d, *J* = 4.8 Hz, 1H), 7.14 (t, *J* = 4.4 Hz, 1H), 1.41 (s, 9H); ^13^C NMR (100 MHz, CDCl_3_) δ 160.51 (s), 133.89 (s), 132.91 (s), 129.53 (s), 127.97 (s), 84.49 (s), 26.34 (s).

**Synthesis of *Tert*-butyl 3-methylbenzo[b]thiophene-2-carboperoxoate**

A solution of Benzo[b]thiophene-2-carboxylic acid (1 mmol, 193 mmol), SOCl_2_ (2 m mol, 146 uL) in dry benzene (3 mL) stirred 1.5 h at 120 ^o^C. Afterward, the solution was evaporated under vacuum. Anhydrous TBHP (2 mL), dry CH_2_Cl_2_ (3 mL) were added to the system stirred 12 h at 50 ^o^C. The solution was extracted with ethyl acetate (3×20 mL), the combined organic layers were dried with over anhydrous Na_2_SO_4_. The solvent was evaporated under vacuum and the crude product was purified by silica gel column chromatography to give the desired product **(1y)** (isolate yield 50%).

*tert*-Butyl 3-methylbenzo[b]thiophene-2-carboperoxoate **(1y)**: ^1^H NMR (400 MHz, CDCl_3_) δ 7.87–7.82 (m, 2H), 7.51–7.42 (m, 2H), 1.43 (s, 9H); ^13^C NMR (100 MHz, CDCl_3_) δ 161.73 (s), 142.21 (s), 140.61 (s), 139.80 (s), 127.67 (s), 124.80 (s), 123.91 (s), 122.77 (s), 122.64 (s), 84.41 (s), 26.41 (s), 13.52 (s).

**Synthesis of ^13^C-labeled *Tert*-Butyl Peroxy Benzoate**

A solution of benzoic acid (0.82 mmol, 100 mg), SOCl_2_ (2 mmol, 146 uL) in dry benzene (3 mL) stirred 1.5 h at 120 ^o^C. Afterward, the solution was evaporated under vacuum. Anhydrous TBHP (2 mL), dry CH_2_Cl_2_ (3 mL) were added to the system stirred 12 h at 50 ^o^C. The solution was extracted with ethyl acetate (3×20 mL), the combined organic layers were dried with over anhydrous Na_2_SO_4_. The solvent was evaporated under vacuum and the crude product was purified by silica gel column chromatography to give the desired product (isolate yield 73%).

*^13^C-labeled tert*-Butyl Peroxy Benzoate: ^1^H NMR (400 MHz, CDCl_3_) δ 7.97–7.93 (m, 2H), 7.59 (t, *J* = 7.4 Hz, 1H), 7.50 (t, *J* = 8.4 Hz, 2H), 1.42 (s, 9H); ^13^C NMR (100 MHz, CDCl_3_) δ 164.62 (s), 162.50 (s), 133.51 (s), 129.26 (s), 128.79 (s), 84.16 (s), 26.38 (s).

**4. Experimental procedure and characterization data for products**

**A typical experimental procedure**: A solution of *tert*-Butyl peresters (0.5 mmol), KO*^t^*Bu (2.5 equiv, 140 mg) in amide compounds (5 equiv/10 equiv) were stirred in a sealed tube under an atmosphere of air at 40 °C for 3 h. After being cooled to room temperature, the reaction mixture were extracted with ethyl acetate (20 mL). Afterward, the solution was evaporated under vacuum. The residue was purified by preparative thin-layer chromatography (TLC) on silica gel with petroleum ether and ethyl acetate (5% triethylamine) to achieve the pure product.

***N,N*-dimethylbenzamide (3a).** Brown oil; Isolated yield 70%; ^1^H NMR (400 MHz, CDCl_3_) δ 7.40 (m, 5H), 3.11 (s, 3H), 2.98 (s, 3H); ^13^C NMR (100 MHz, CDCl_3_) δ 171.70 (s), 136.33 (s), 129.57 (s), 128.39 (s), 127.07 (s), 39.64 (s), 35.38 (s); IR (neat) 3140, 3029, 2932, 2361, 1716, 1634, 1579, 1506, 1485, 1446, 1398, 1265, 1217, 1085, 792, 736, 714, 639, 557 cm^-1^; HRMS ( ESI) m/z calcd for C_9_H_15_N_2_O 167.1179, found [M+NH_4_]^+^ 167.1176.

***N*-methylbenzamide (3b).** White solid; Isolated yield 89%; Mp 72–74 ^o^C; ^1^H NMR (400 MHz, CDCl_3_) δ 7.78–7.76 (m, 2H), 7.48 (t, *J* = 7.0 Hz, 1H), 7.41 (t, *J* = 7.6 Hz, 2H), 6.43 (br s, NH), 3.00 (d, *J* = 4.8 Hz, 3H); ^13^C NMR (100 MHz, CDCl_3_) δ 168.43 (s), 134.71 (s), 131.45 (s), 128.64 (s), 126.95 (s), 26.95 (s); IR (neat) 3152, 1646, 1579, 1548, 1489, 1402, 1310, 712, 695, 529 cm^-1^; HRMS ( ESI) m/z calcd for C_8_H_10_NO 136.0757, found [M+H]^+^ 136.0756.

**Benzamide (3c).** White solid; Isolated yield 99%; Mp 122–125 ^o^C; ^1^H NMR (400 MHz, CDCl_3_) δ 7.82 (dd, *J* = 6.6, 1.0 Hz, 2H), 7.47–7.43 (m, 2H), 6.17 (br s, NH); ^13^C NMR (100 MHz, CDCl_3_) δ 169.70 (s), 133.51 (s), 132.14 (s), 128.76 (s), 127.47 (s); IR (neat) 3153, 1698, 1402, 1152, 527 cm^-1^; HRMS (ESI) m/z calcd for C_7_H_7_NNaO 144.0420, found [M+Na]^+^ 144.0419.

**Benzohydrazide (3d)**. White solid; Isolated yield 53%; Mp 106–108 ^o^C; ^1^H NMR (400 MHz, CDCl_3_) δ 7.81 (br s, NH), 7.76 (d, *J* = 7.2 Hz, 2H), 7.52 (t, *J* = 7.4 Hz, 1H), 7.44 (t, *J* = 7.4 Hz, 2H), 3.76 (br s, NH); ^13^C NMR (100 MHz, CDCl_3_) δ 168.85 (s), 132.73 (s), 132.03 (s), 128.83 (s), 127.00 (s); IR (neat) 3152, 2378, 1672, 1402, 1167, 1115, 527, 419 cm^-1^; HRMS ( ESI) m/z calcd for C_7_H_9_N_2_O 137.0709, found [M+H]^+^ 137.0785.

***N*-ethylbenzamide (3e).** Light yellow oil; Isolated yield 73%; ^1^H NMR (400 MHz, CDCl_3_) δ 8.50–8.48 (m, 2H), 8.15 (t, *J* = 7.4 Hz, 1H), 8.07 (t, *J* = 7.6 Hz, 2H), 7.58 (br s, NH), 4.18–4.11 (m, 2H), 1.91 (t, *J* = 7.2 Hz, 3H); ^13^C NMR (100 MHz, CDCl_3_) δ 167.65 (s), 134.74 (s), 131.21 (s), 128.40 (s), 126.94 (s), 34.91 (s), 14.81 (s); IR (neat) 3155, 2978, 2935, 2876, 2361, 1657, 1579, 1560, 1439, 1402, 1310, 1183, 1148, 930, 869, 805, 712, 527 cm^-1^; HRMS ( ESI) m/z calcd for C_9_H_15_N_2_O 167.1179, found [M+NH_4_]^+^ 167.1176.

***N*-benzylbenzamide (3f)**. Brown solid; Isolated yield 77%; Mp 102–104 ^o^C; ^1^H NMR (400 MHz, CDCl_3_) δ 7.81–7.78 (m, 2H), 7.52–7.48 (m, 1H), 7.44 –7.40 (m, 2H), 7.36–7.35 (m, 4H), 7.32–7.28 (m, 1H), 6.49 (br s, NH), 4.64 (d, *J* = 5.6 Hz, 2H); ^13^C NMR (100 MHz, CDCl_3_) δ 167.49 (s), 138.29 (s), 134.48 (s), 131.69 (s), 128.92 (s), 128.73 (s), 128.05 (s), 127.76 (s), 127.08 (s), 44.26 (s); IR (neat) 3153, 2365, 1642, 1541, 1402, 1157, 1078, 990, 729, 691, 669, 460 cm^-1^; HRMS ( ESI) m/z calcd for C_14_H_14_NO 212.1070, found [M+H]^+^ 212.1068.

**2-methylbenzamide (3h).** White solid; Isolated yield 82%; Mp 138–139 ^o^C; ^1^H NMR (400 MHz, CDCl_3_) δ 7.46 (dd, *J* = 7.4, 1.0 Hz, 1H), 7.34 (td, *J* = 7.6, 2.8 Hz, 1H), 7.25–7.20 (m, 2H), 5.80 (br s, NH) 2.50 (s, 3H); ^13^C NMR (100 MHz, CDCl_3_) δ 172.04 (s), 136.54 (s), 135.22 (s), 131.39 (s), 130.48 (s), 127.08 (s), 125.90 (s), 20.14 (s); IR (neat) 3152, 1638, 1402, 1139, 779, 637, 531 cm^-1^; HRMS ( ESI) m/z calcd for C_8_H_10_NO 136.0757, found [M+H]^+^ 136.0756.

**4-(*tert*-butyl)benzamide (3i)**. White solid; Isolated yield 73%; Mp 161–163 ^o^C; ^1^H NMR (400 MHz, CDCl_3_) δ 7.76 (d, *J* = 8.8 Hz, 2H), 7.47 (d, *J* = 8.4 Hz, 2H), 6.11 (br s, NH), 5.91 (br s, NH), 1.34 (s, 9H); ^13^C NMR (100 MHz, CDCl_3_) δ 169.52 (s), 155.74 (s), 130.54 (s), 127.36 (s), 125.71 (s), 35.12 (s), 31.28 (s); IR (neat) 3152, 2367, 1649, 1612, 1402, 1128, 669, 527 cm^-1^; HRMS ( ESI) m/z calcd for C_11_H_16_NO 178.1226, found [M+H]^+^ 178.1223.

**2-methoxybenzamide (3j)**. White solid; Isolated yield 73%; Mp 126–127 ^o^C; ^1^H NMR (400 MHz, CDCl_3_) δ 8.22 (dd, *J* = 7.8, 1.8 Hz, 1H), 7.74 (br s NH), 7.51–7.46 (m, 1H), 7.11–7.07 (m, 1H), 7.00 (d, *J* = 8.4 Hz, 1H), 5.99 (br s, NH), 3.98 (s, 3H); ^13^C NMR (100 MHz, CDCl_3_) δ 167.18 (s), 157.94 (s), 133.53 (s), 132.72 (s), 121.39 (s), 120.89 (s), 111.48 (s), 56.06 (s); IR (neat) 3152, 2363, 1698, 1620, 1402, 1150, 527 cm^-1^; HRMS ( ESI) m/z calcd for C_8_H_9_NNaO_2_ 174.0526, found [M+Na]^+^ 174.0534.

**3-methoxybenzamide (3k).** White solid; Isolated yield 56%; Mp 133–136 ^o^C; ^1^H NMR (400 MHz, CDCl_3_) δ 7.40 (s, 1H), 7.37–7.31 (m, 2H), 7.09–7.06 (m, 1H), 6.14 (br s, NH), 5.87 (br s, NH), 3.86 (s, 3H); ^13^C NMR (100 MHz, CDCl_3_) δ 169.37 (s), 160.00 (s), 134.90 (s), 129.76 (s), 119.29 (s), 118.45 (s), 112.72 (s), 55.60 (s); IR (neat) 3153, 1664, 1629, 1584, 1400, 1251, 1131, 1033, 796, 688, 527 cm^-1^; HRMS ( ESI) m/z calcd for C_8_H_10_NO_2_ 152.0706, found [M+H]^+^ 152.0706.

**4-methoxybenzamide (3l).** White solid; Isolated yield 54%; Mp 168–170 ^o^C; ^1^H NMR (400 MHz, CDCl_3_) δ 7.79 (d, *J* = 9.2 Hz, 2H), 6.94 (d, *J* = 8.8 Hz, 2H), 5.90 (br s, NH), 3.86 (s, 3H); ^13^C NMR (100 MHz, CDCl_3_) δ 168.96 (s), 162.76 (s), 129.43 (s), 125.68 (s), 113.96 (s), 35.58 (s); IR (neat) 3392, 3153, 1646, 1620, 1519, 1400, 1310, 1256, 1182, 1025, 818, 598, 529 cm^-1^; HRMS ( ESI) m/z calcd for C_8_H_9_NNaO_2_ 174.0526, found [M+Na]^+^ 174.0533.

**4-methoxy-*N*-methylbenzamide (3m).** Brown solid; Isolated yield 63%; Mp 121–123 ^o^C; ^1^H NMR (400 MHz, CDCl_3_) δ 7.73 (d, *J* = 8.8 Hz, 2H), 6.91 (d, *J* = 8.8 Hz, 2H), 6.23 (br s, NH), 3.84 (s, 3H), 2.99 (d, *J* = 4.8 Hz, 3H); ^13^C NMR (100 MHz, CDCl_3_) δ 167.93 (s), 162.16 (s), 128.73 (s), 127.05 (s), 113.83 (s), 55.51 (s), 26.91 (s); IR (neat) 3360, 3140, 3008, 2846, 1625, 1558, 1510, 1454, 1403, 1316, 1256, 1187, 1156, 1033, 848, 770, 606 cm^-1^; HRMS ( ESI) m/z calcd for C_9_H_11_NNaO_2_ 188.0682, found [M+Na]^+^ 188.0682.

***N*-ethyl-4-methoxybenzamide (3n).** Yellow oil; Isolated yield 55%; ^1^H NMR (400 MHz, CDCl_3_) δ 7.75 (d, *J* = 9.2 Hz, 2H), 6.88 (d, *J* = 8.8 Hz, 2H), 6.50 (br s, NH), 3.82 (s, 3H), 3.49–3.42 (m, 2H), 1.22 (t, *J* = 7.2 Hz, 2H); ^13^C NMR (100 MHz, CDCl_3_) δ 167.16 (s), 162.03 (s), 128.74 (s), 127.10 (s), 113.67 (s), 55.41 (s), 34.91 (s), 14.96 (s); IR (neat) 3152, 2976, 2840, 2050, 1636, 1541, 1508, 1443, 1402, 1314, 1256, 1180, 1146, 1109, 1031, 870, 770, 632, 610, 535 cm^-1^; HRMS ( ESI) m/z calcd for C_10_H_13_NNaO_2_ 202.0839, found [M+Na]^+^ 202.0838.

**4-methoxy-*N,N*-dimethylbenzamide (3o)**. Light yellow oil; Isolated yield 55%; ^1^H NMR (400 MHz, CDCl_3_) δ 7.40 (d, *J* = 8.8 Hz, 2H), 6.90 (d, *J* = 8.4 Hz, 2H), 3.83 (s, 3H), 3.06 (m, 6H); ^13^C NMR (100 MHz, CDCl_3_) δ 171.60 (s), 160.66 (s), 129.20 (s), 128.46 (s), 113.61 (s), 55.40 (s), 39.88 (br s), 35.64 (br s); IR (neat) 3137, 3008, 2937, 2840, 2551, 2048, 1638, 1491, 1400, 1300, 1252, 1174, 1113, 1082, 1029, 919, 800, 717, 687, 595, 486 cm^-1^; HRMS ( ESI) m/z calcd for C_10_H_14_NO_2_ 180.1019, found [M+H]^+^ 180.1018.

**3-nitrobenzamide (3p).** White solid; Isolated yield 80%; Mp 150–152 ^o^C; ^1^H NMR (400 MHz, CD_3_COCD_3_) δ 8.75 (t, *J* = 2.0 Hz, 1H), 8.40 (ddd, *J* = 8.2, 2.3, 1.0 Hz, 1H), 8.38–8.35 (m, 1H), 7.89 (br s, NH), 7.80 (t, *J* = 8.0 Hz, 1H), 7.06 (br s, NH); ^13^C NMR (100 MHz, CD_3_COCD_3_) δ 166.79 (s), 149.25 (s), 136.96 (s), 134.45 (s), 130.81 (s), 126.69 (s), 123.18(s); IR (neat) 3152, 2363, 1672, 1402, 1111, 721, 669, 527, cm^-1^; HRMS ( ESI) m/z calcd for C_7_H10N_3_O_3_ 184.0717, found [M+NH_4_]^+^ 184.0732.

***N*-methyl-3-nitrobenzamide (3q).** Green solid; Isolated yield 60%; Mp 182–184 ^o^C; ^1^H NMR (400 MHz, CD_3_COCD_3_) δ 8.69 (t, *J* = 1.8 Hz, 1H), 8.38 (ddd, *J* = 8.2, 2.3, 1.0 Hz, 1H), 8.32–8.29 (m, 1H), 8.12 (br s, NH), 7.78 (t, *J* = 8.0 Hz, 1H), 2.95 (d, *J* = 4.8 Hz, 3H); ^13^C NMR (100 MHz, CD_3_COCD_3_) δ 165.62 (s), 149.23 (s), 137.47 (s), 134.04 (s), 130.81 (s), 126.37 (s), 122.70 (s), 26.84 (s); IR (neat) 3153, 1655, 1402, 1450, 727, 1455, 671, 527 cm^-1^; HRMS ( ESI) m/z calcd for C_8_H_8_N_2_KO_3_ 219.0167, found [M+K]^+^ 219.0158.

**1-naphthamide (3s).** White solid; Isolated yield 88%; Mp 205–207 ^o^C; ^1^H NMR (400 MHz, CD_3_COCD_3_) δ 8.49–8.46 (m, 1H), 7.99 (d, *J* = 8.4 Hz, 1H), 7.95–7.92 (m, 1H), 7.73 (dd, *J* = 6.8, 1.2 Hz, 1H), 7.58–7.49 (m, 3H), 7.37 (br s, NH), 6.91 (br s, NH); ^13^C NMR (100 MHz, CD_3_COCD_3_) δ 171.39 (s), 135.50 (s), 134.72 (s), 131.28 (s), 131.00 (s), 129.03 (s), 127.41 (s), 126.98 (s), 126.82 (s), 126.08 (s), 125.65 (s); IR (neat) 3153, 2363, 1661, 1402, 1120, 814, 779, 527, 503, 419 cm^-1^; HRMS ( ESI) m/z calcd for C_11_H_13_N_2_O 189.1022, found [M+NH_4_]^+^ 189.1023.

**2-naphthamide (3t).** White solid; Isolated yield 70%; Mp 202–203 ^o^C; ^1^H NMR (400 MHz, CD_3_COCD_3_) δ 8.53 (s, 1H), 8.05–7.95 (m, 4H), 7.68 (br s, NH), 6.83 (br s, NH); ^13^C NMR (100 MHz, CD_3_COCD_3_) δ 169.03 (s), 135.69 (s), 133.63 (s), 132.73 (s), 129.81 (s), 128.81 (s), 128.70 (s), 128.54 (s), 128.41 (s), 127.46 (s), 125.29 (s); IR (neat) 3140, 1657, 1402, 1116, 915, 839, 872, 787, 639 cm^-1^; HRMS ( ESI) m/z calcd for C_11_H_10_NO 172.0757, found [M+H]^+^ 172.0756.

***N,N*-dimethyl-1-naphthamide (3u)**. Brown oil; Isolated yield 31%; ^1^H NMR (400 MHz, CDCl_3_) δ 7.88–7.86 (m, 2H), 7.80–7.78 (m, 1H), 7.54–7.46 (m, 3H), 7.42 (dd, *J* = 7.2, 1.2 Hz, 1H), 3.26 (s, 3H), 2.81 (s, 3H); ^13^C NMR (100 MHz, CDCl_3_) δ 171.01 (s), 134.82 (s), 133.55 (s), 129.56 (s), 129.12 (s), 128.50 (s), 127.06 (s), 126.45 (s), 125.29 (s), 124.94 (s), 123.97 (s), 38.97 (s), 34.97 (s); IR (neat) 3153, 2371, 1655, 1560, 1508, 1402, 1274, 1260, 1176, 1122, 1060, 867, 803, 781, 634, 527, 511, 472 cm^-1^; HRMS ( ESI) m/z calcd for C_13_H_14_NO 200.1070, found [M+H]^+^ 200.1068.

**Furan-2-carboxamide (3v)**. Brown solid; Isolated yield 56%; Mp 136–137 ^o^C; ^1^H NMR (400 MHz, CDCl_3_) δ 7.48 (s, 1H), 7.17 (d, *J* = 3.2 Hz, 1H), 6.52 (dd, *J* = 3.4, 1.7 Hz, 1H), 6.28 (br s, NH), 6.05 (br s, NH); ^13^C NMR (100 MHz, CDCl_3_) δ 160.29 (s), 147.54 (s), 144.54 (s), 115.34 (s), 112.46 (s); IR (neat) 3153, 2361, 1661, 1627, 1523, 1402, 1109, 885, 710, 595 cm^-1^; HRMS ( ESI) m/z calcd for C_5_H_6_NO_2_ 112.0393, found [M+H]^+^ 112.0395.

**Thiophene-2-carboxamide (3w).** White solid; Isolated yield 57%; Mp 184–186 ^o^C; ^1^H NMR (400 MHz, CD_3_COCD_3_) δ 7.73 (dd, *J* = 3.8, 1.0 Hz, 1H), 7.68 (dd, *J* = 5.2, 1.2 Hz, 1H), 7.43 (br s, NH), 7.12 (dd, *J* = 4.8, 3.6 Hz, 1H), 6.67 (br s, NH); ^13^C NMR (100 MHz, CD_3_COCD_3_) δ 163.77 (s), 141.12 (s), 131.42 (s), 129.20 (s), 128.49 (s); IR (neat) 3152, 1649, 1607, 1402, 1243, 1098, 712, 643 cm^-1^; HRMS ( ESI) m/z calcd for C_5_H_6_NOS 128.0165, found [M+H]^+^ 128.0163.

***N,N*-dimethylthiophene-2-carboxamide (3x).** Light yellow oil; Isolated yield 66%; ^1^H NMR (400 MHz, CDCl_3_) δ 7.44 (d, *J* = 5.2 Hz, 1H), 7.35 (d, *J* = 2.8 Hz, 1H), 7.04 (t, *J* = 4.4 Hz, 1H); ^13^C NMR (100 MHz, CDCl_3_) δ 164.38 (s), 137.91 (s), 129.16 (s), 128.80 (s), 126.70 (s), 39.50 (br s), 36.51 (br s); IR (neat) 3118, 2345, 1655, 1400, 1265, 1204, 1183, 1085, 850, 740, 670 cm^-1^; HRMS ( ESI) m/z calcd for C_7_H_9_NOSNa 178.0297, found [M+Na]^+^ 178.0294.

**3-methylbenzo[*b*]thiophene-2-carboxamide (3y)**. White solid; Isolated yield 63%; Mp 194–197 ^o^C; ^1^H NMR (400 MHz, CD_3_COCD_3_) δ 7.95–7.91 (m, 1H), 7.90–7.86 (m, 1H), 7.50–7.44 (m, 2H), 7.01 (br s, NH), 2.70 (s, 3H); ^13^C NMR (100 MHz, CD_3_COCD_3_) δ 165.43 (s), 141.43 (s), 139.64 (s), 135.78 (s), 132.60 (s), 127.22 (s), 125.41 (s), 124.09 (s), 123.35 (s), 13.09 (s); IR (neat) 3152, 1651, 1646, 1616, 1402, 1116, 755, 727, 527 cm^-1^; HRMS ( ESI) m/z calcd for C_10_H_10_NOS 192.0478, found [M+H]^+^ 192.0485.

**^13^C-Labeled Benzamide.** White solid; Isolated yield 55%; Mp 112-116 ^o^C; ^1^H NMR (400 MHz, CDCl_3_) δ 7.84–7.81 (m, 2H), 7.53 (t, *J* = 7.4 Hz, 1H), 7.44 (t, *J* = 7.8 Hz, 2H), 6.32 (br s, NH); ^13^C NMR (100 MHz, CDCl_3_) δ 169.87 (s), 133.81 (s), 133.18 (s), 132.11 (s), 128.73 (d, *J* = 4.1 Hz), 127.46 (d, *J* = 4.1 Hz).

**5. Gram-scale synthesis of Benzamide (3c)**

A solution of *tert*-butyl peroxybenzoate (1 g, 5.15 mmol), KO*^t^*Bu (1.44 g, 12.88 mmol) and formamide (2 mL, 51.5 mmol) were stirred in a sealed tube under an atmosphere of air at 40 °C for 3 h. The reaction mixture was then extracted with ethyl acetate. Afterward, the solution was evaporated under vacuum. The residue was purified by column chromatography (petroleum ether : ethyl acetate = 1:1, v/v, 5% triethylamine was added) with a good yield of 90% (560.0 mg).

**6. References**

[1] H. Zhang, D. Q. Dong, S. H. Hao, Z. L. Wang, *RSC Adv.,* **2016**, *6*, 8465–8468.

[2] W. Wei, C. Zhang, Y. Xu, XB. Wan, *Chem. Commun*., **2011**, *47*, 10827–10829.

**7. Copies of ^1^H and ^13^C NMR spectra**


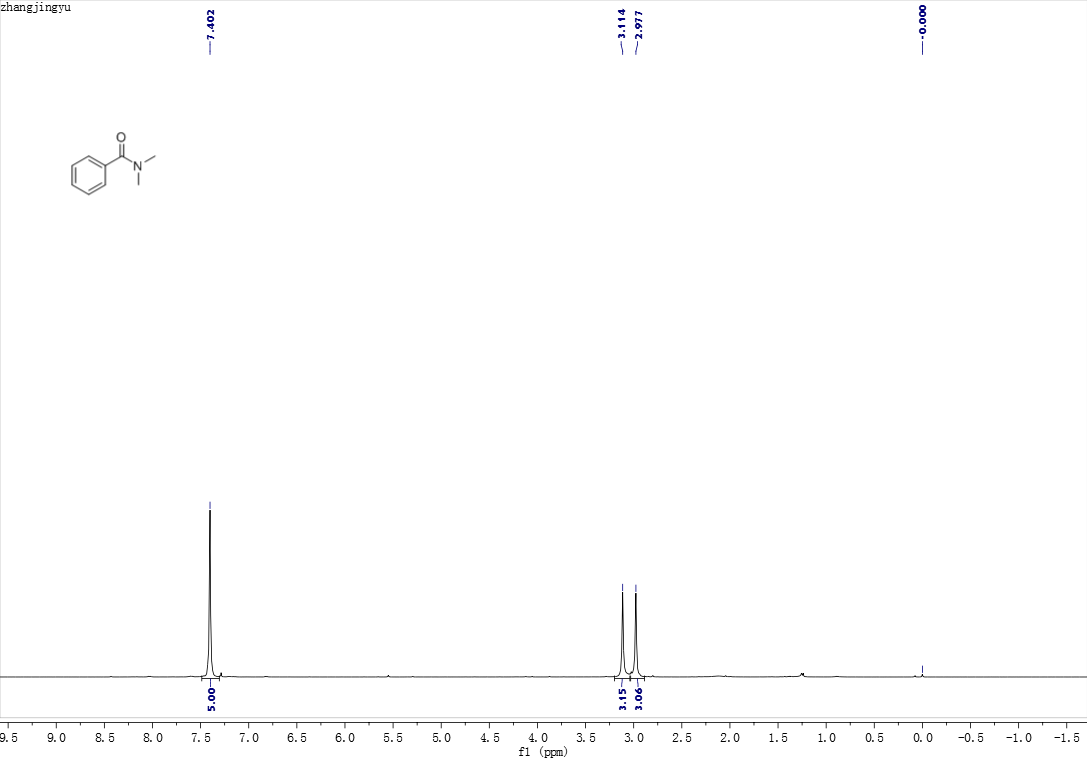


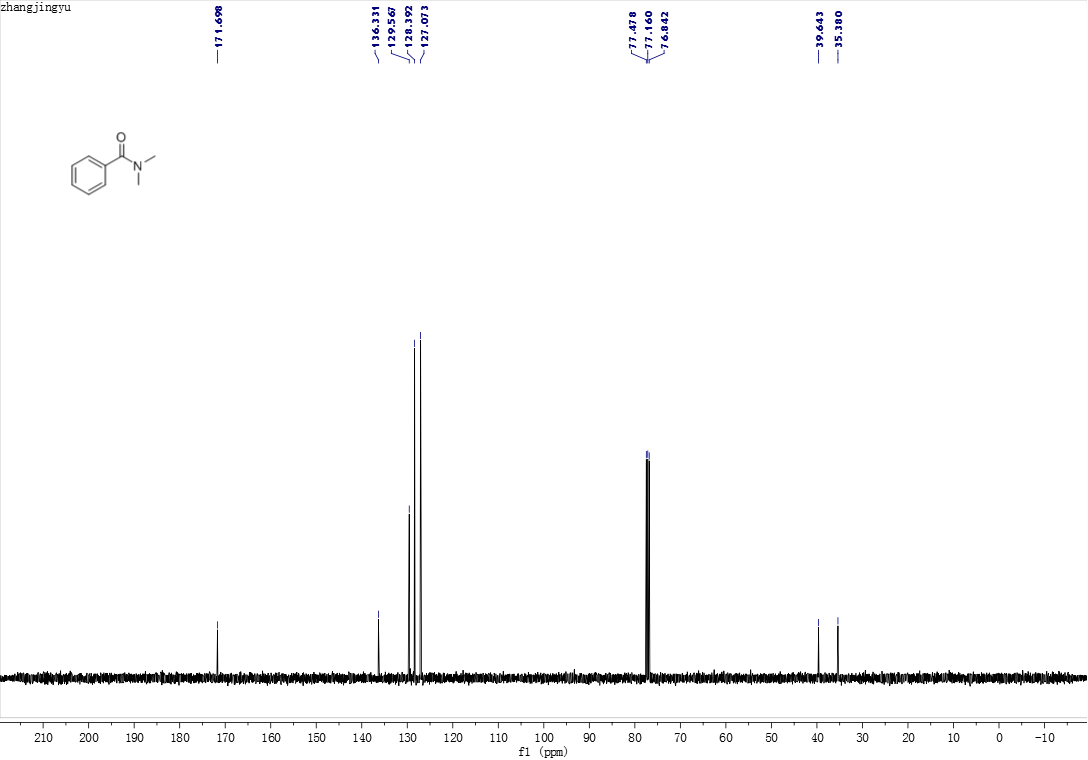


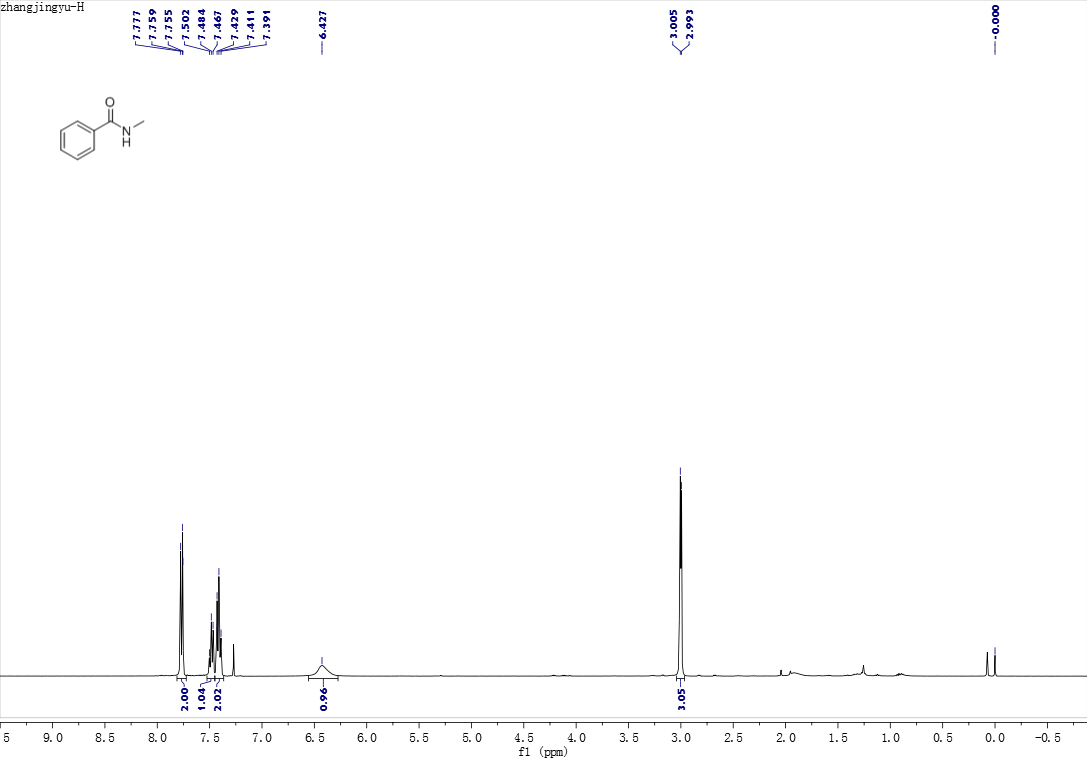


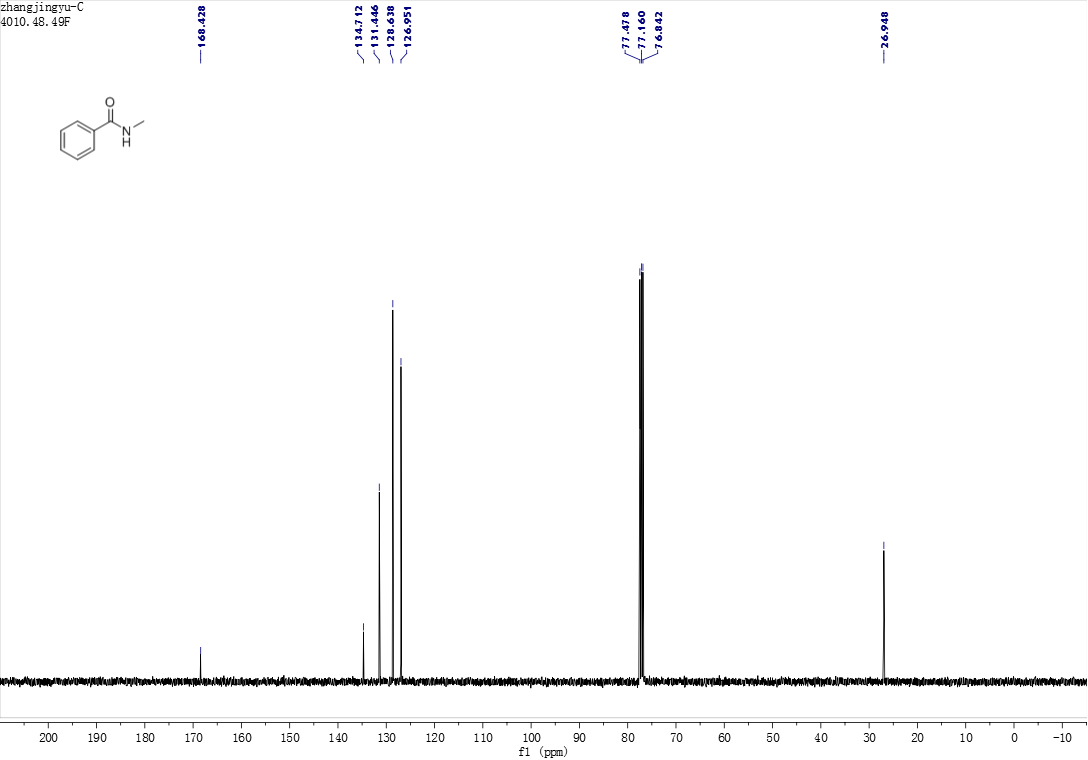


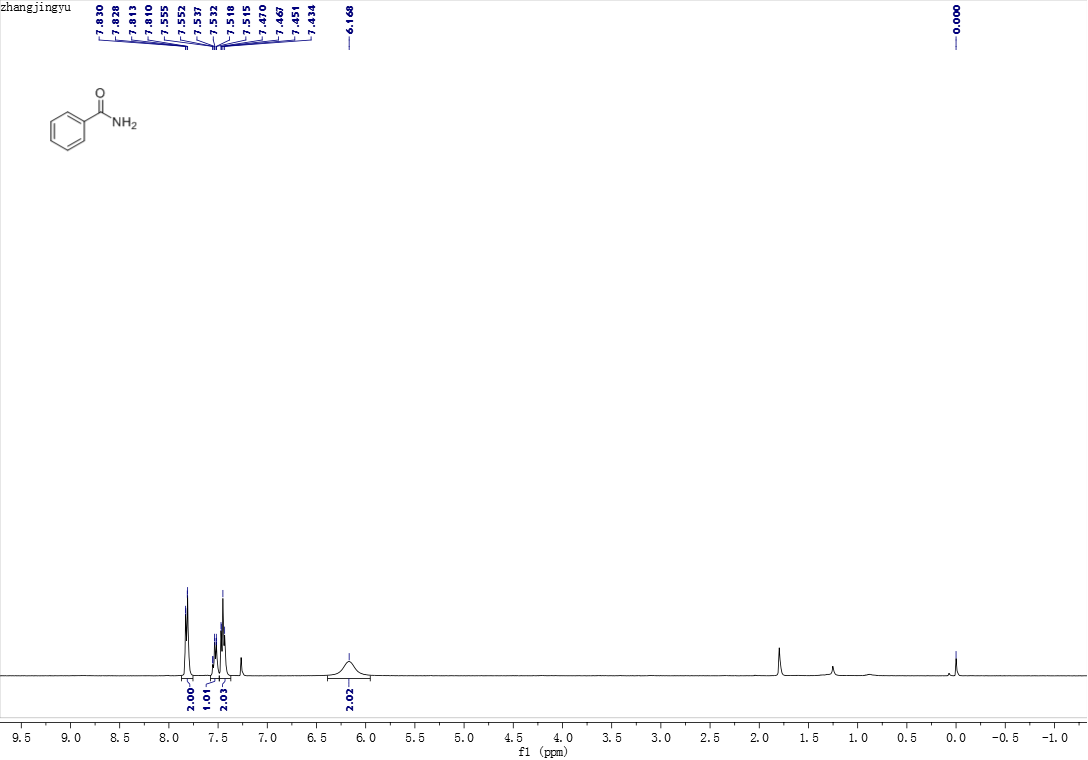


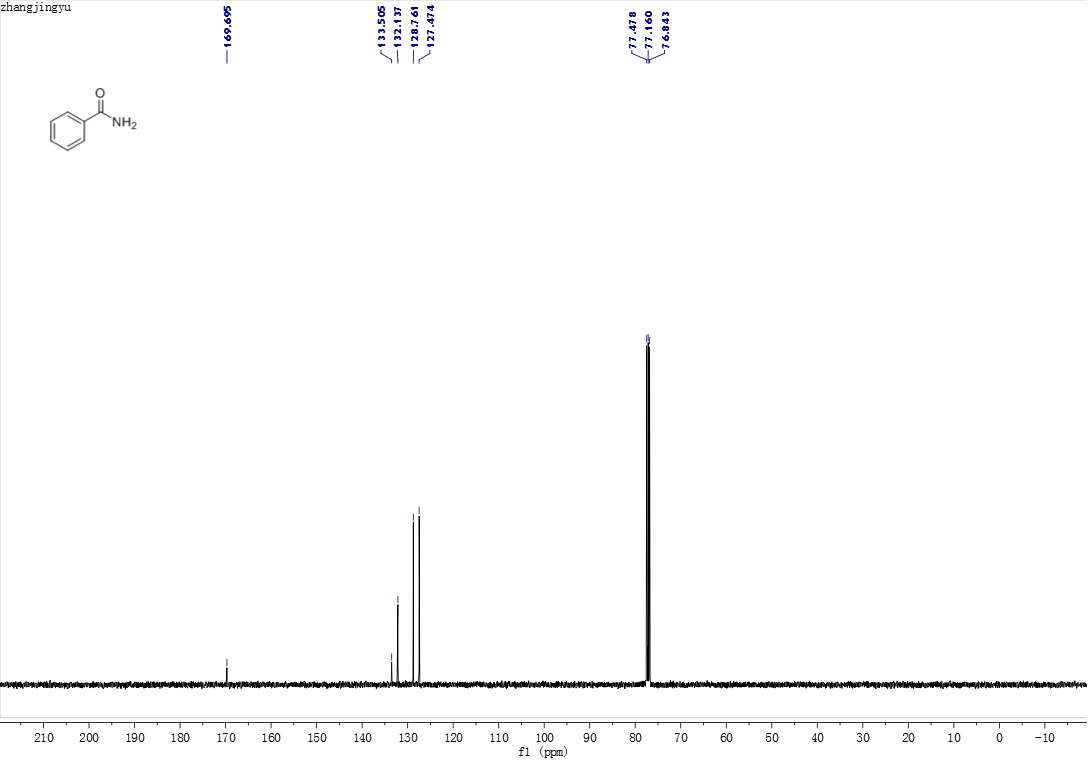


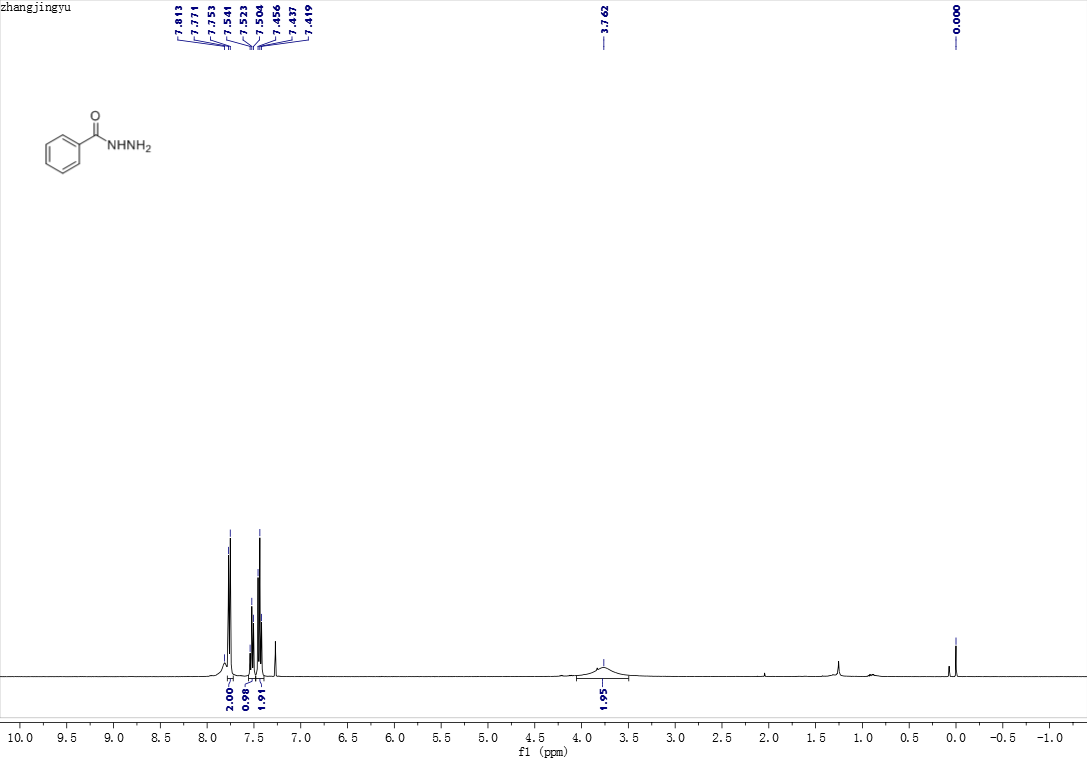


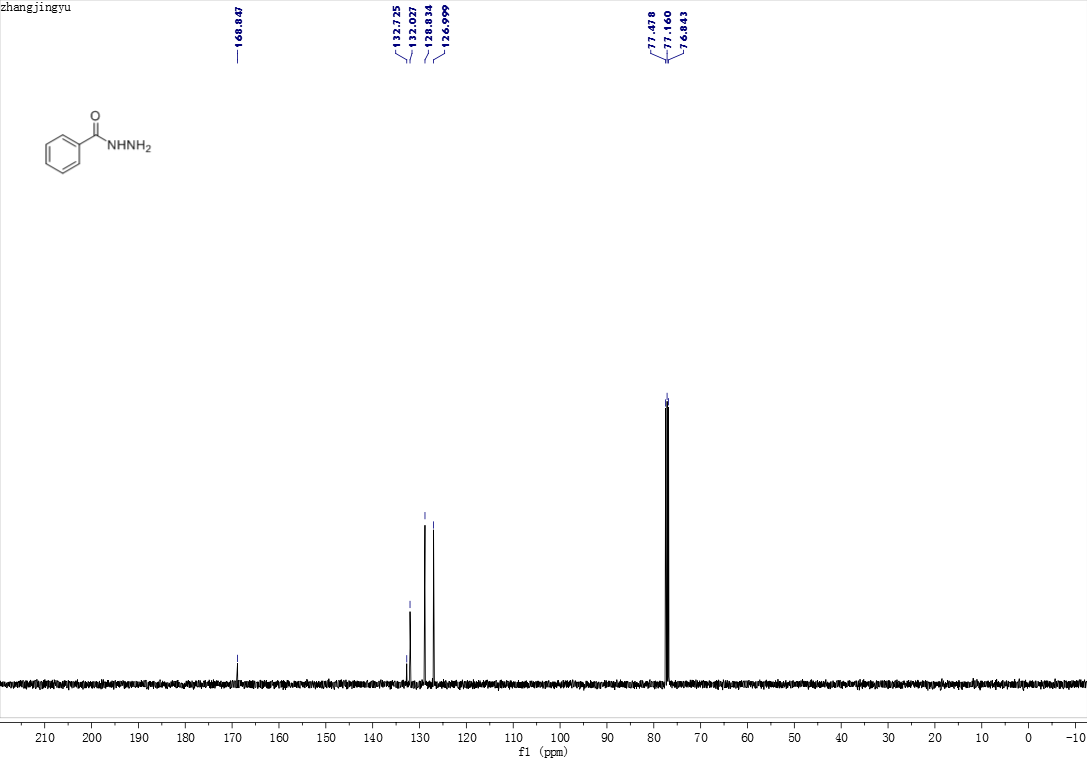


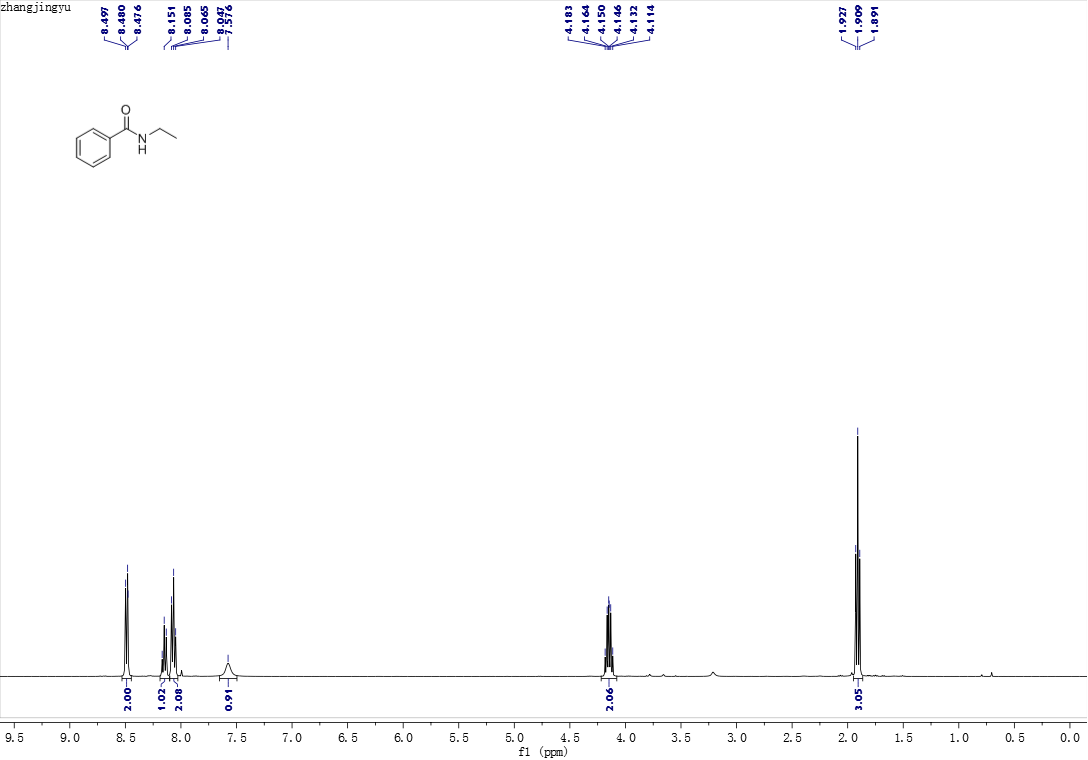


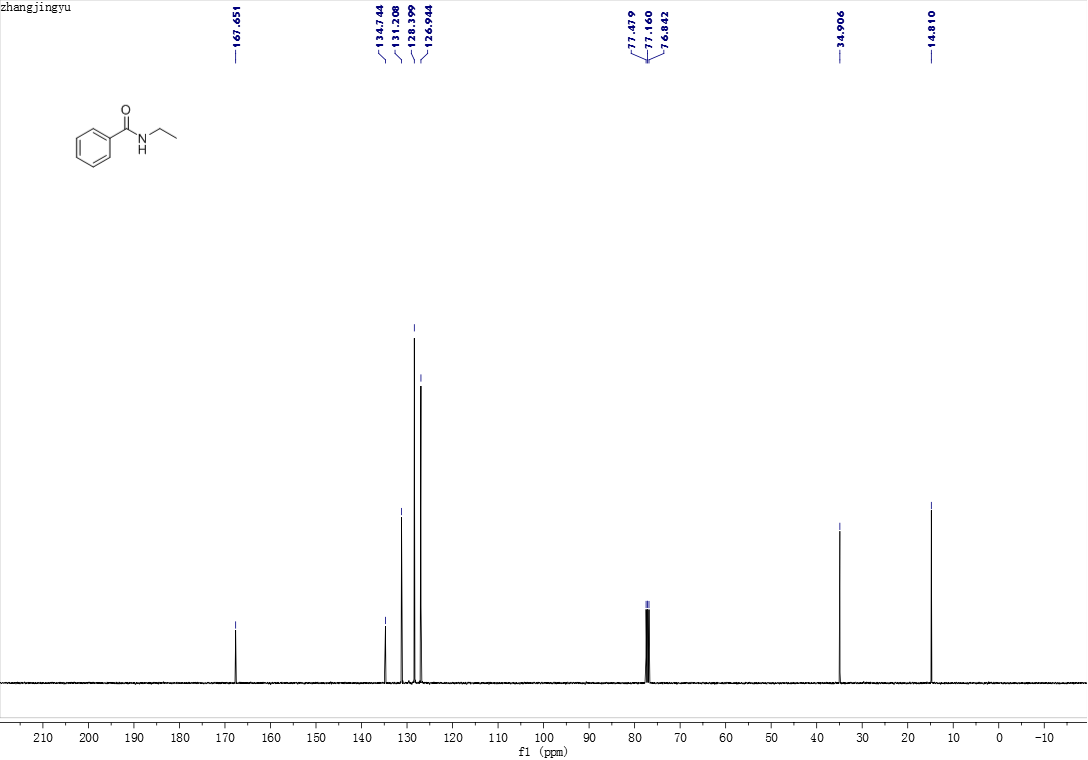


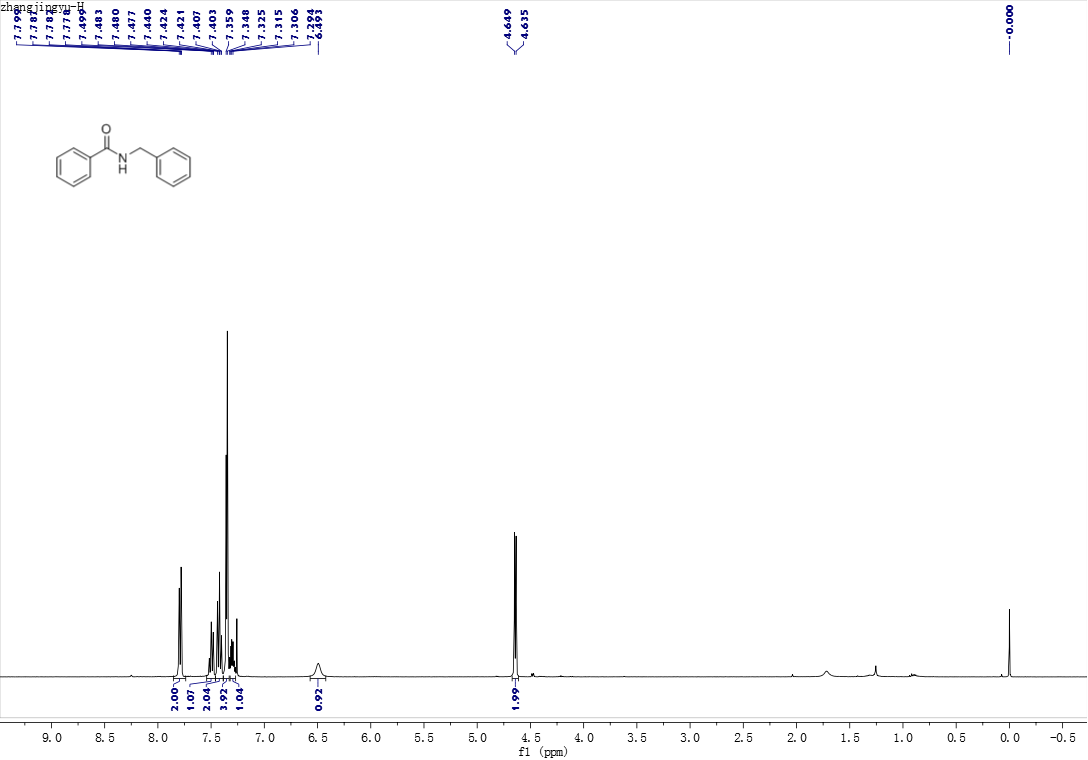


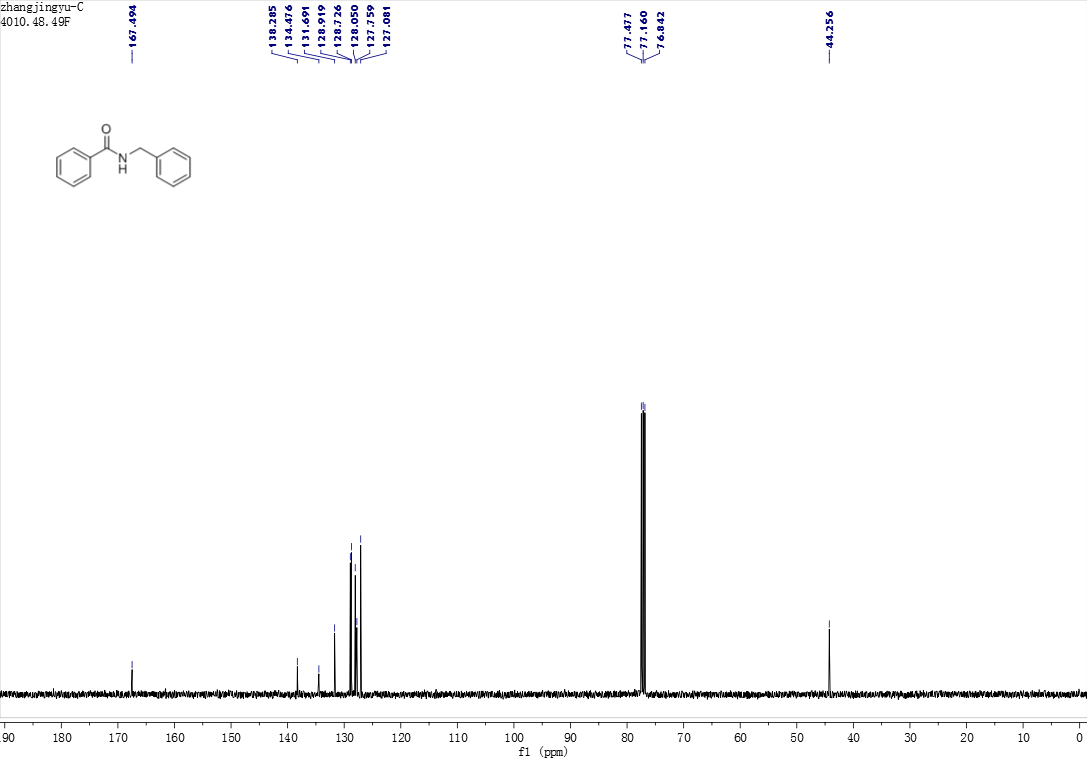


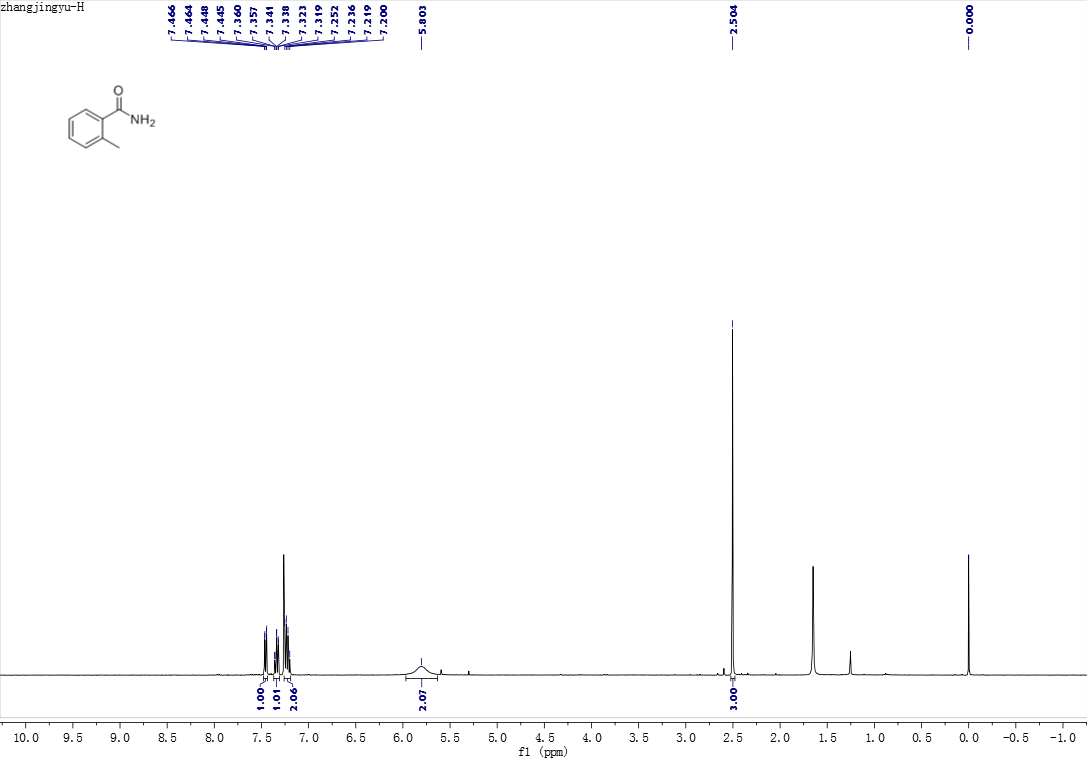


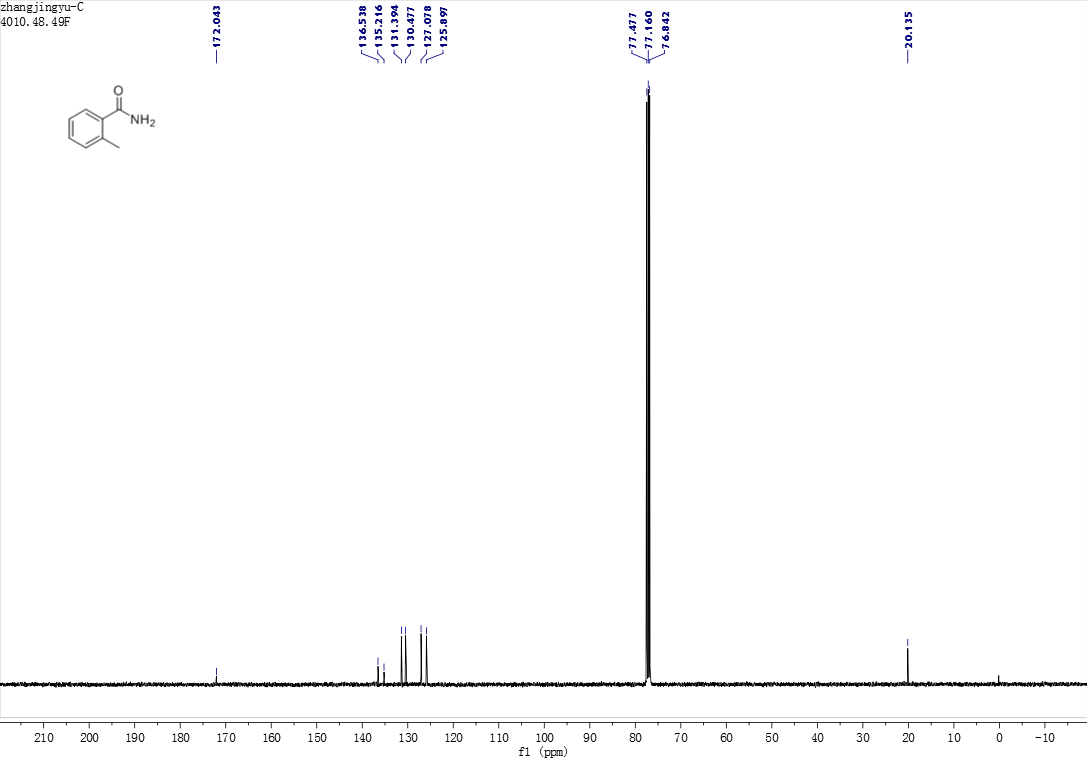


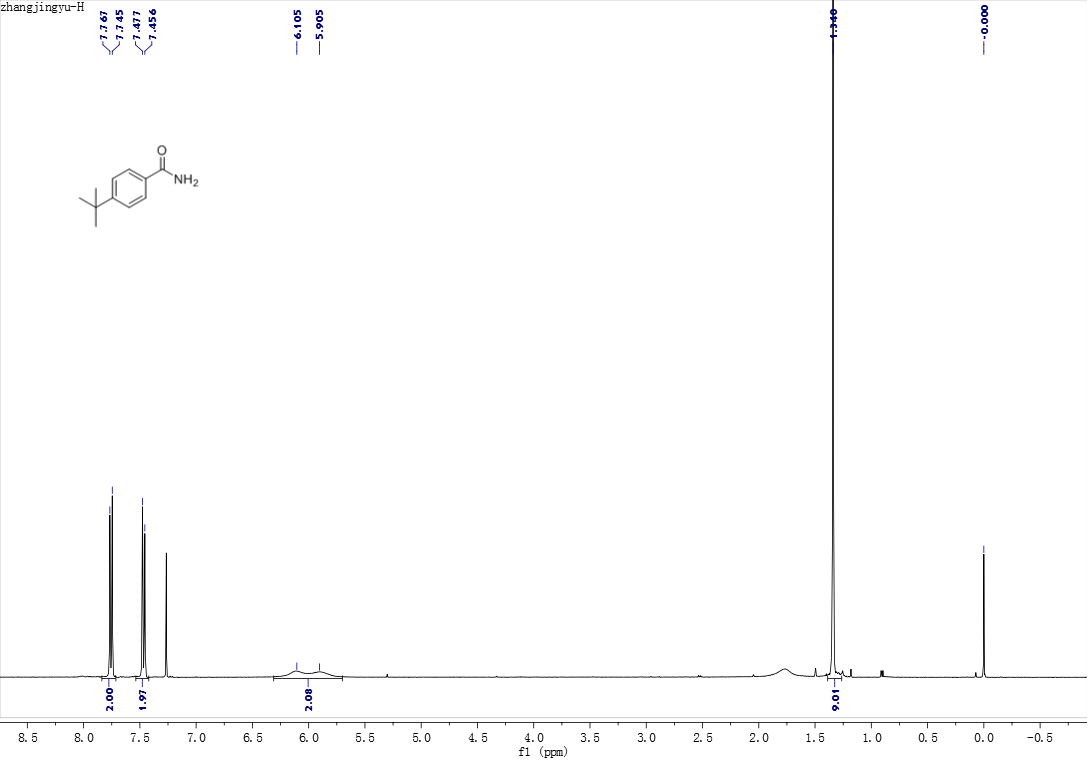

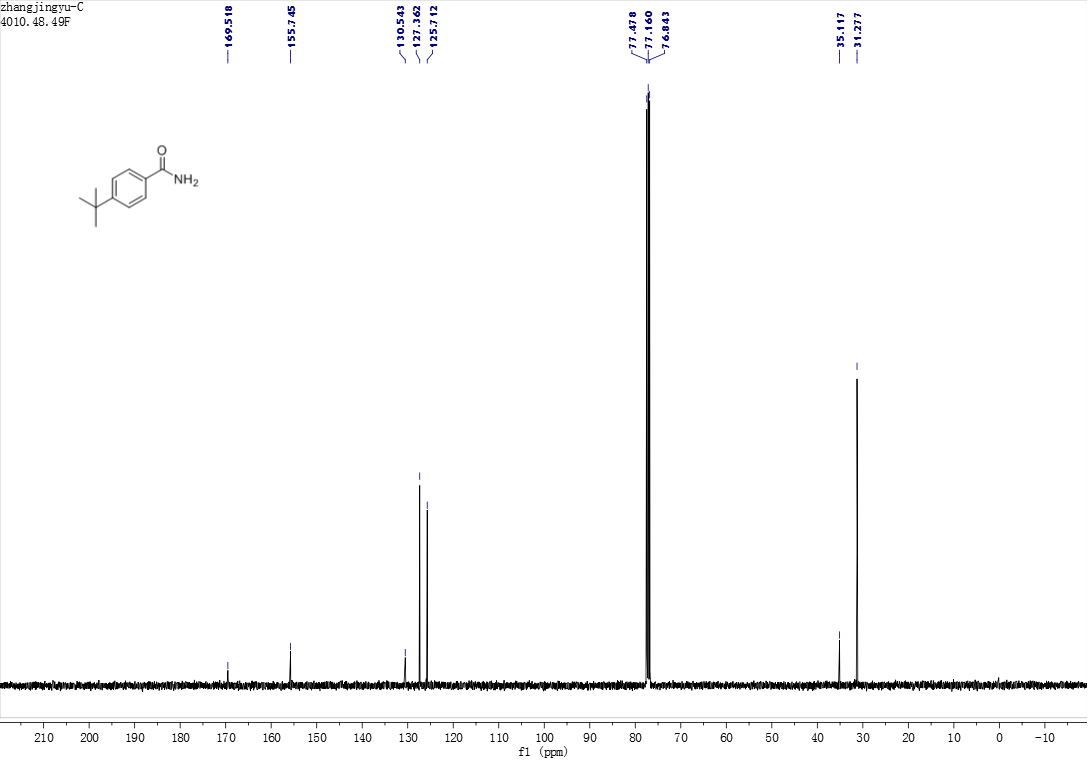


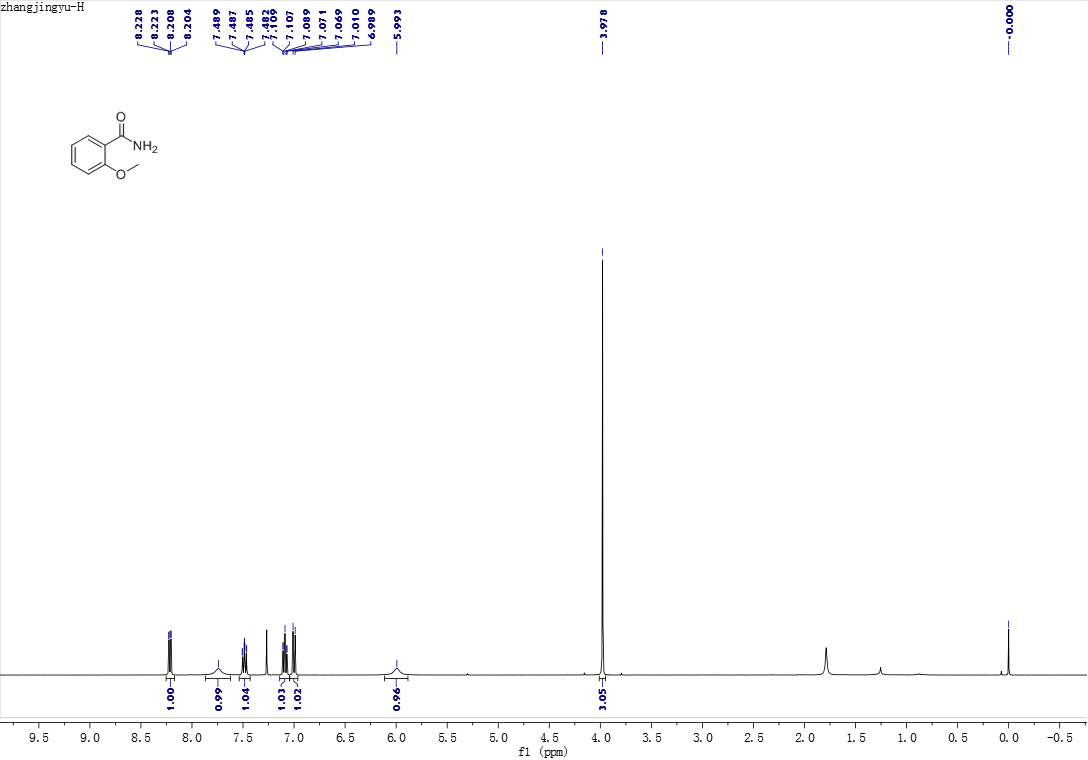


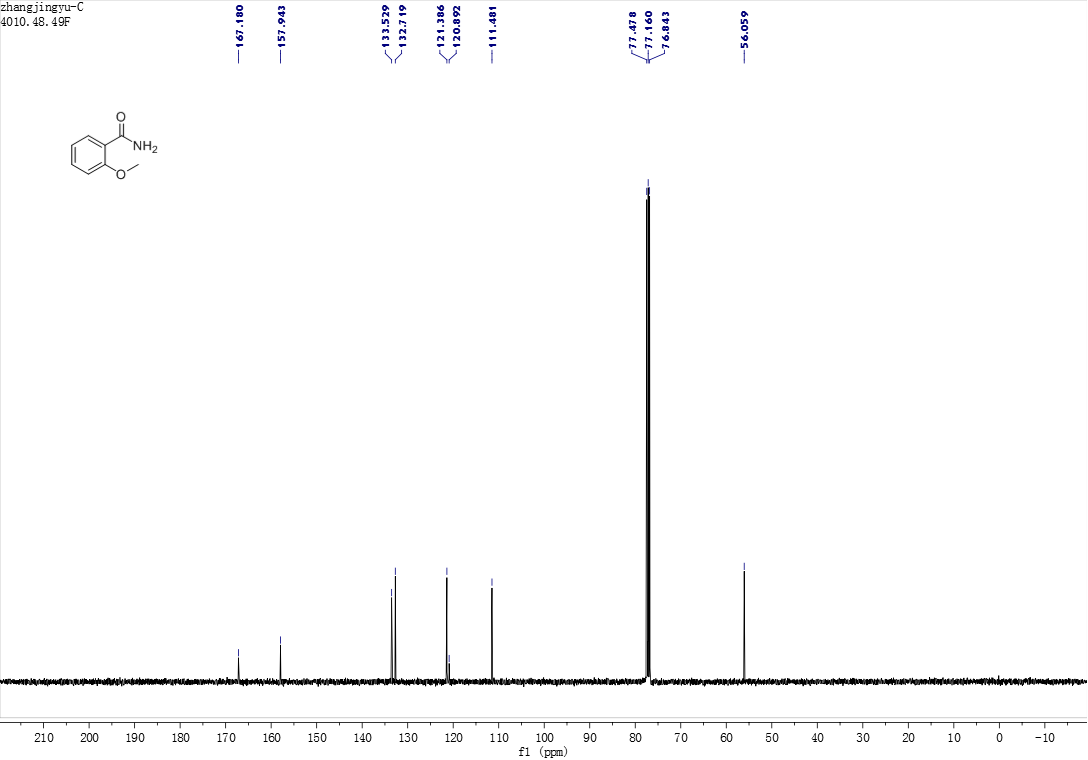


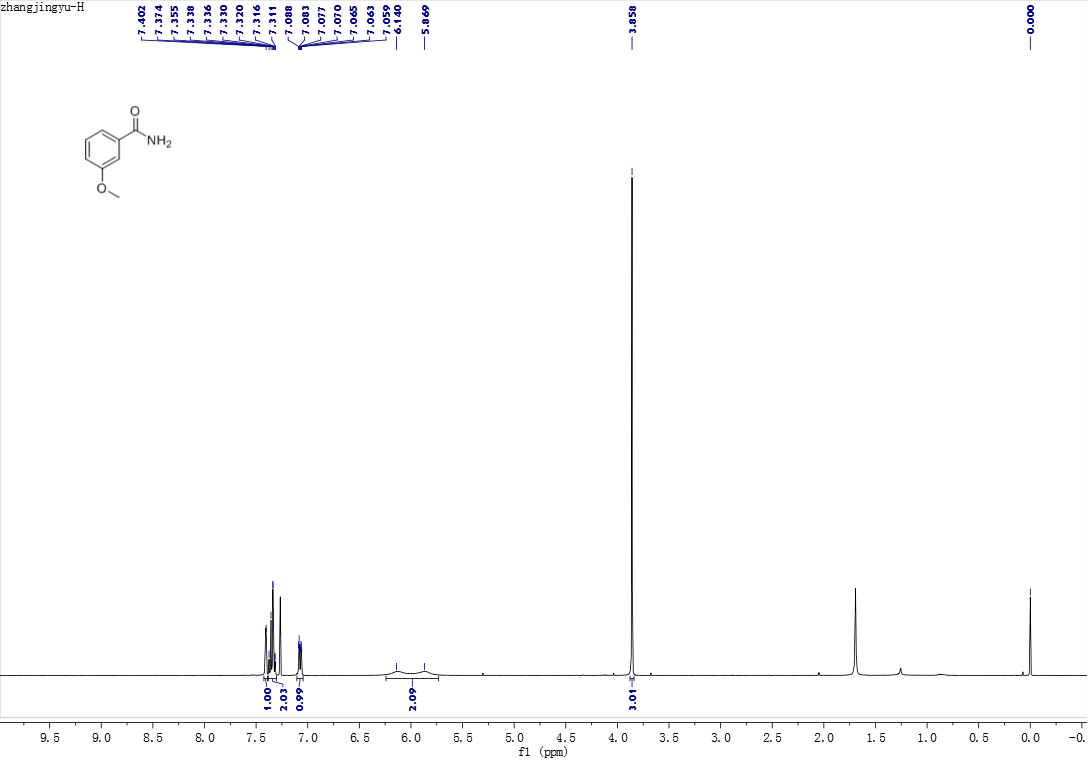


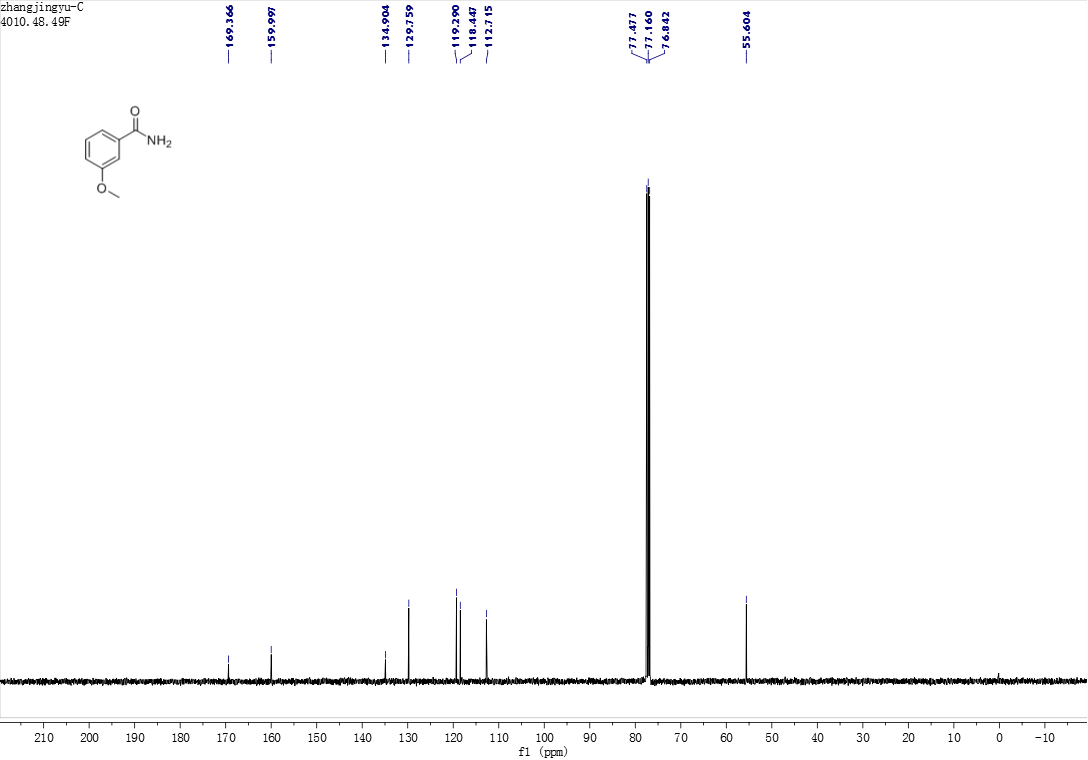


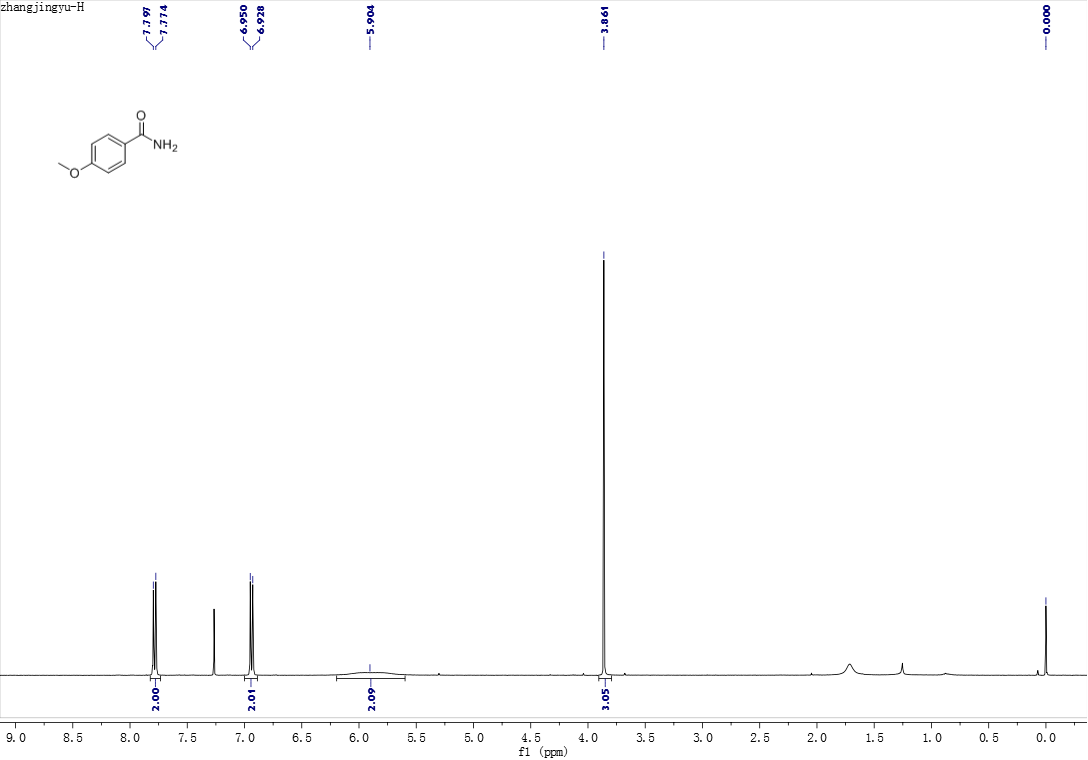


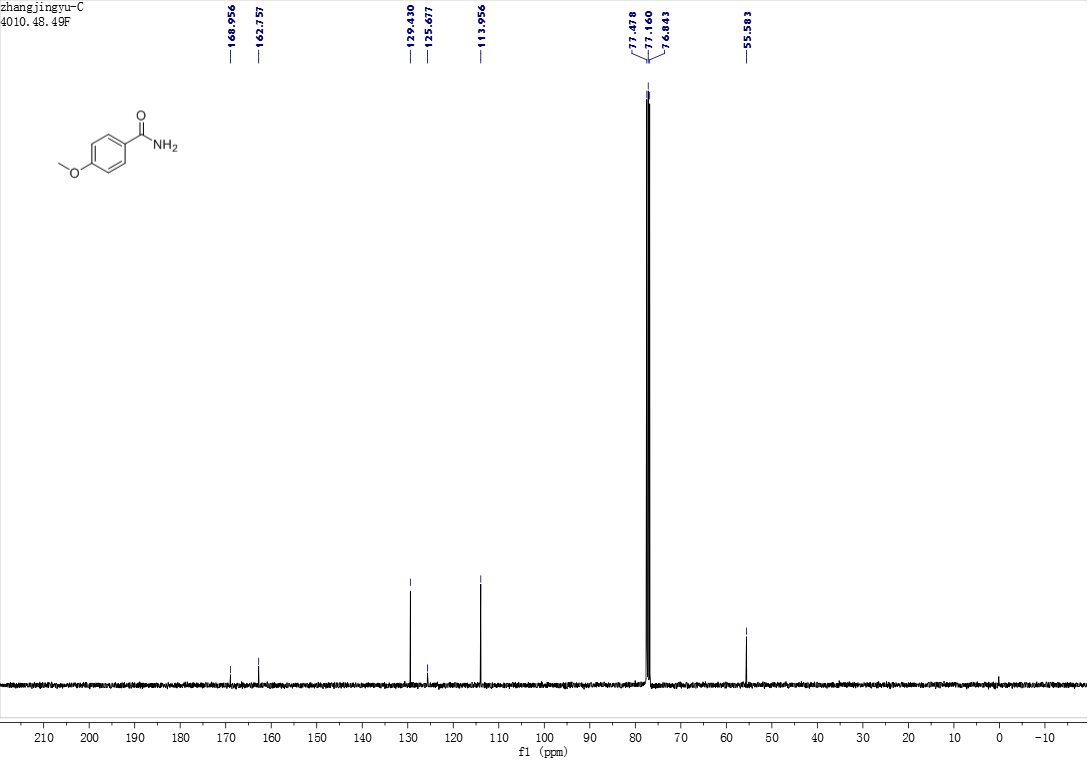


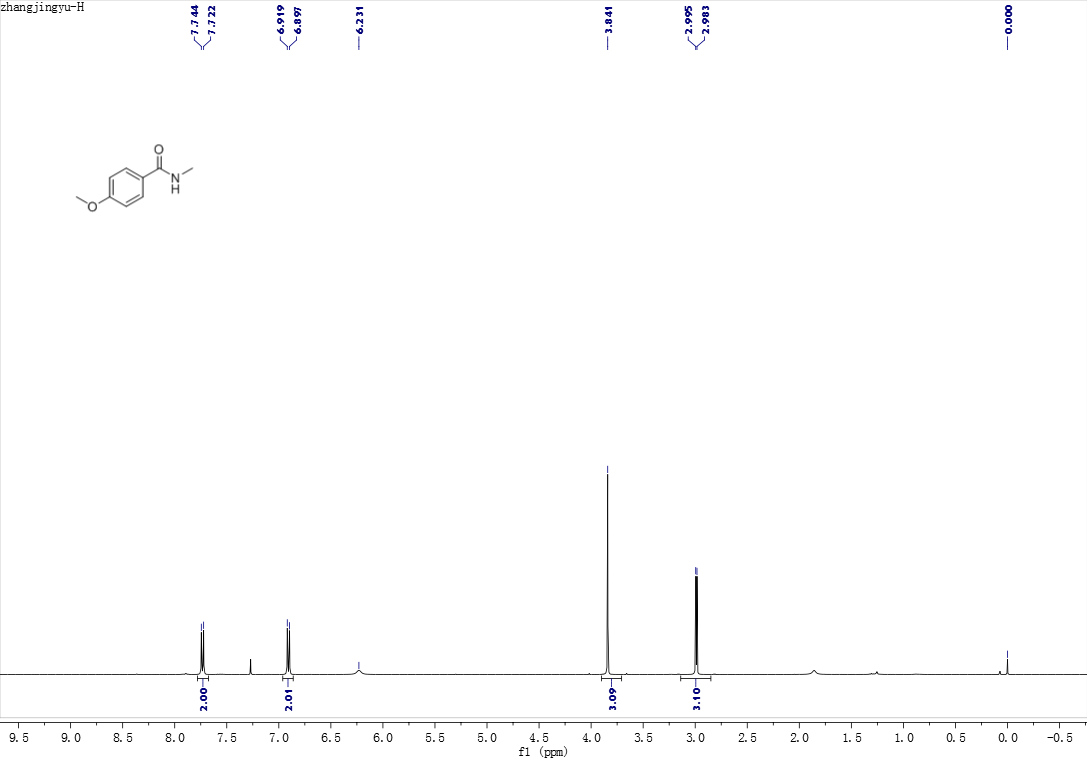


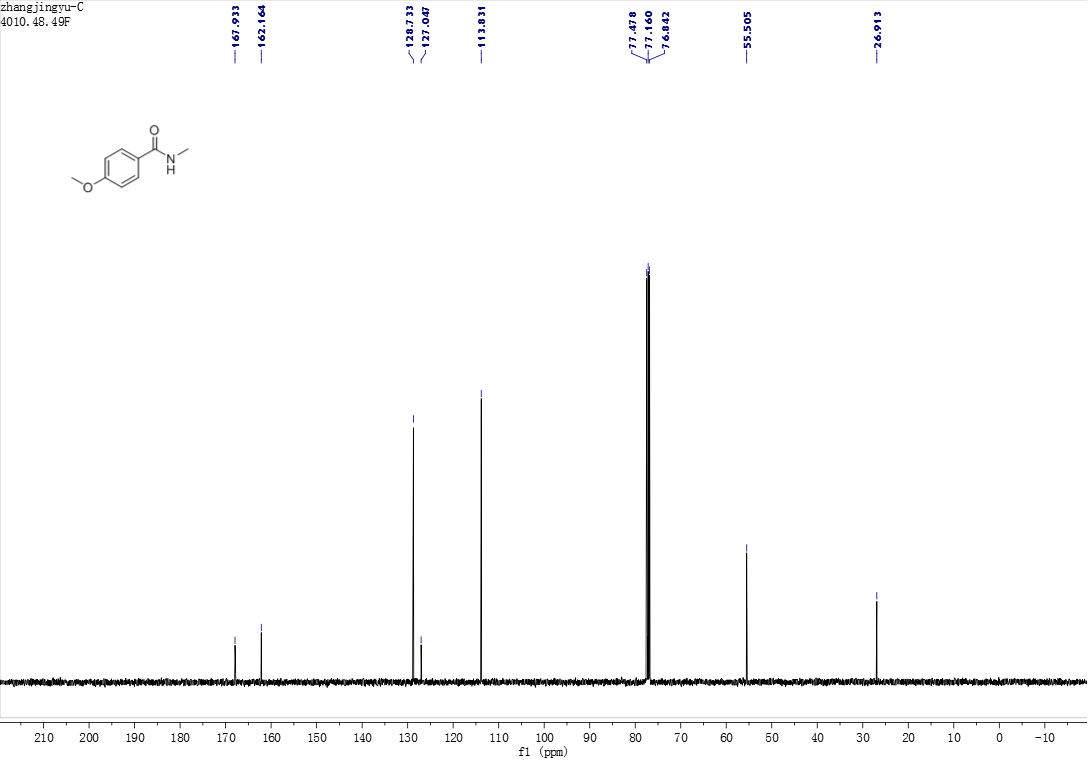


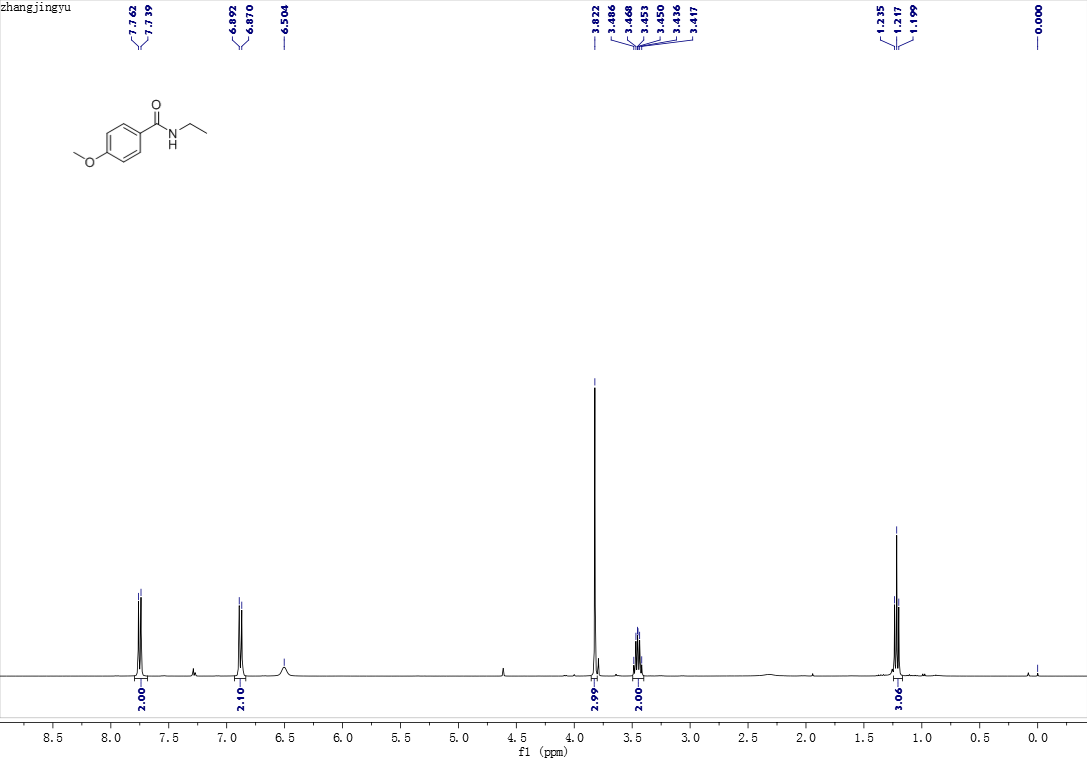


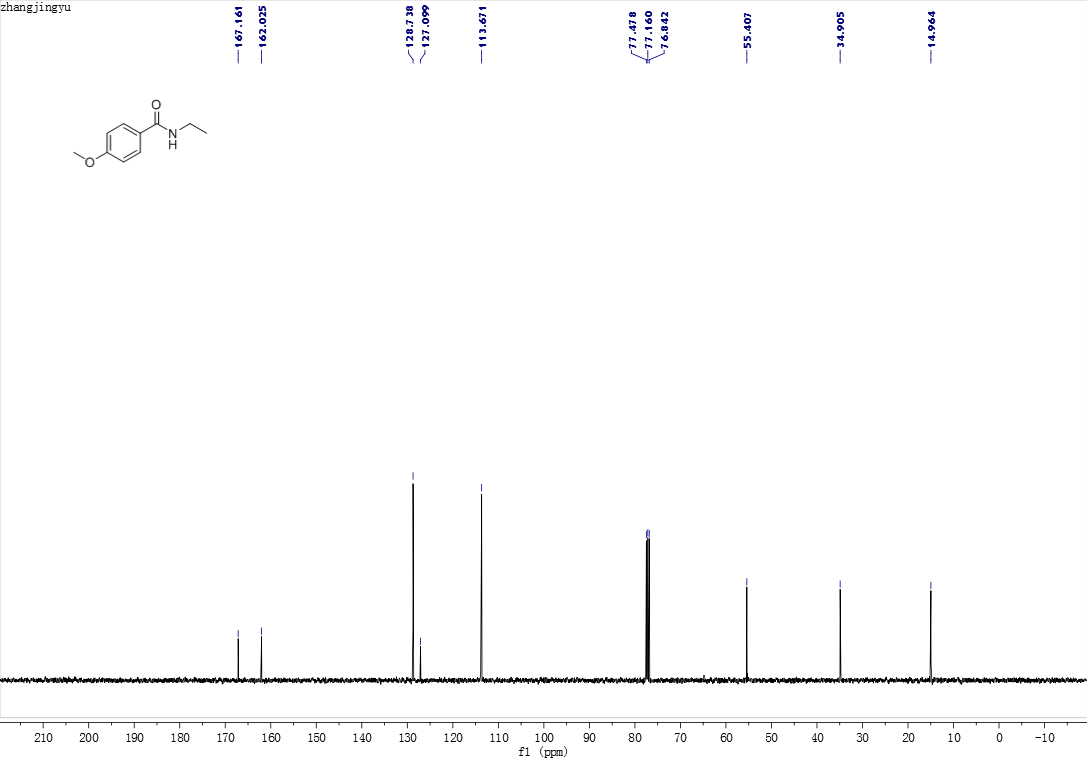


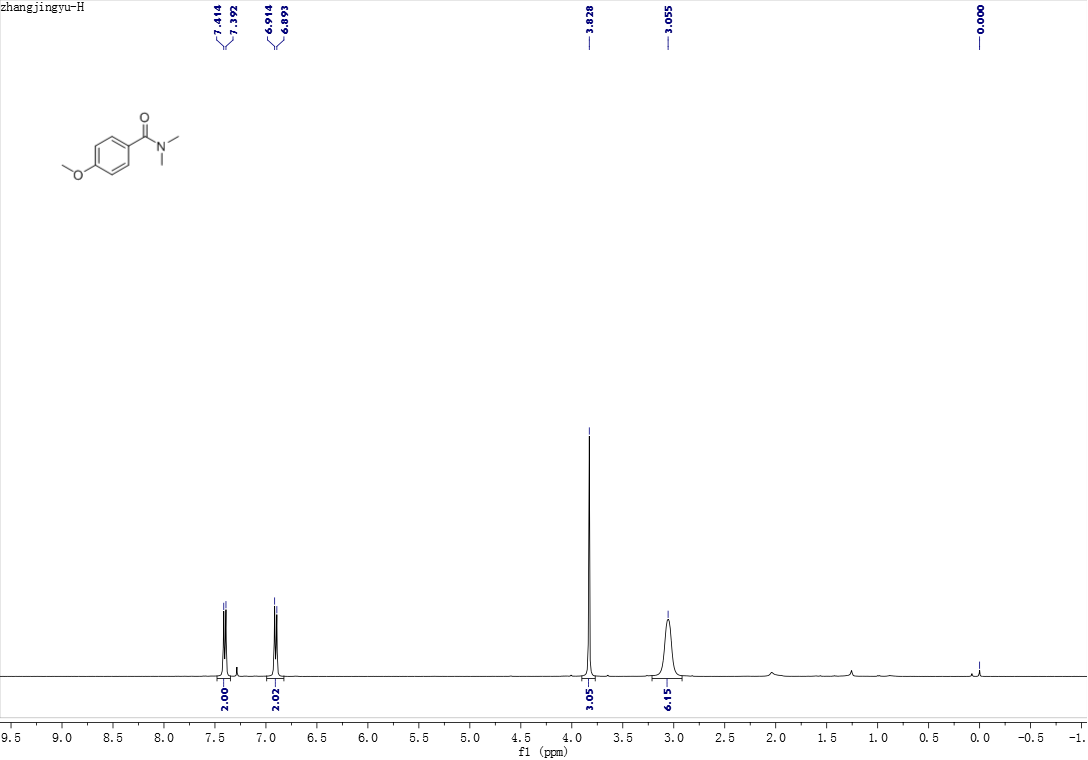


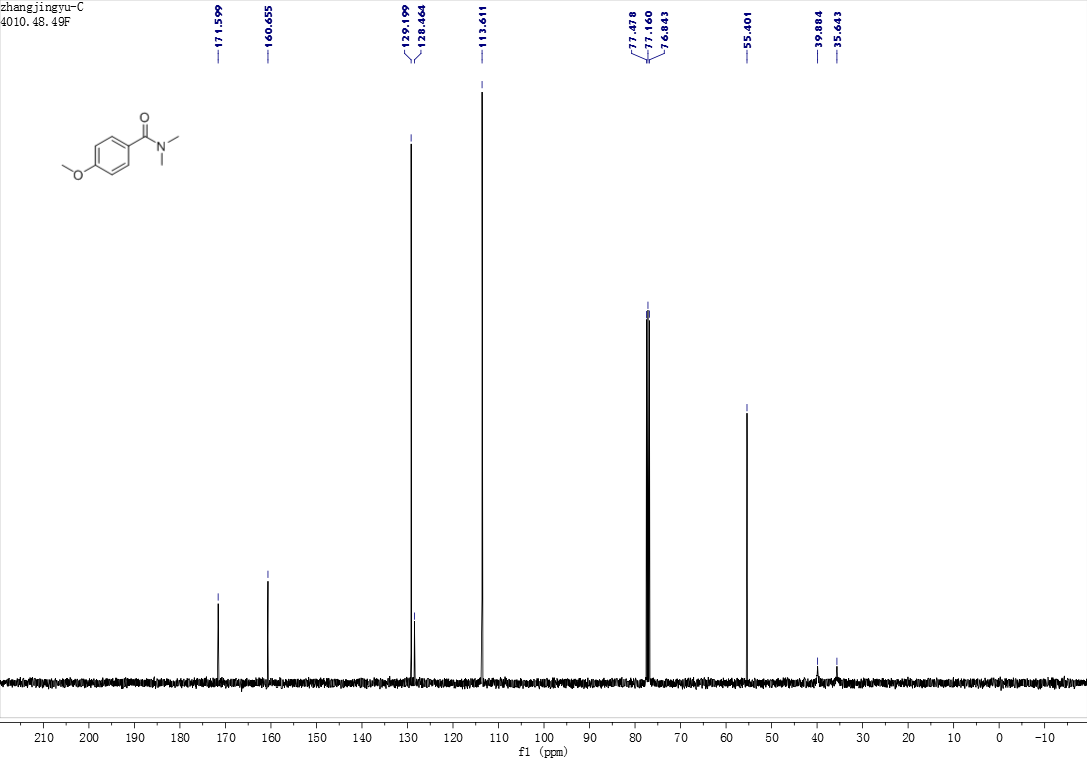


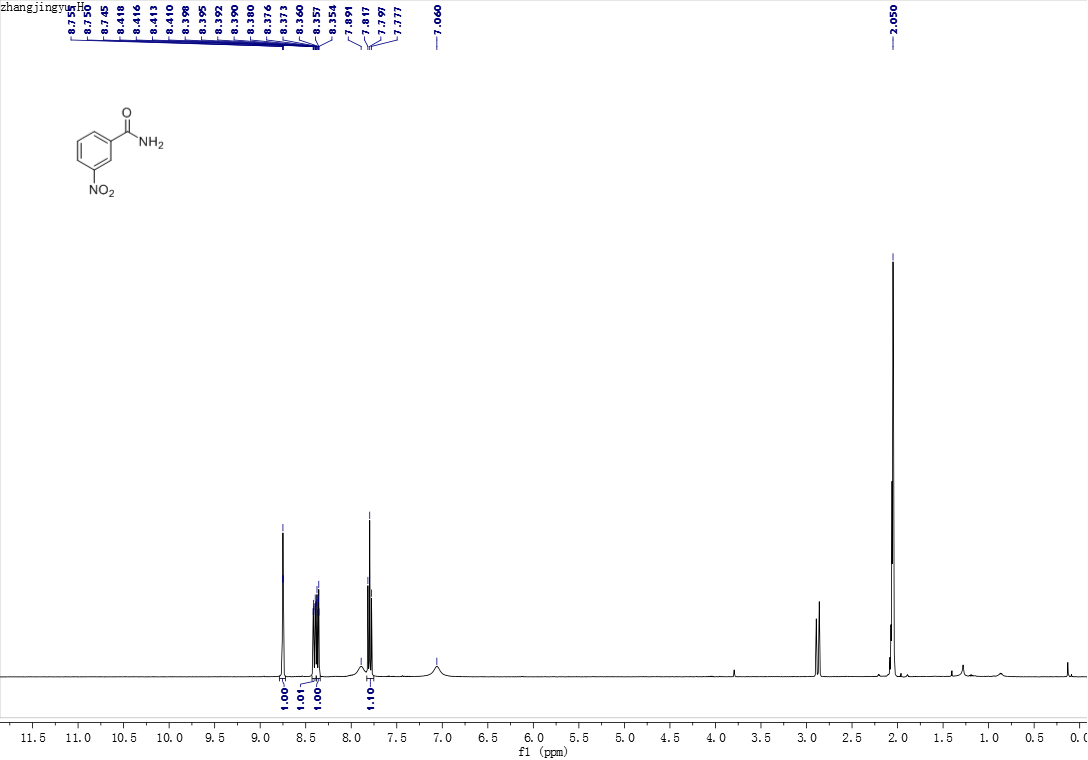


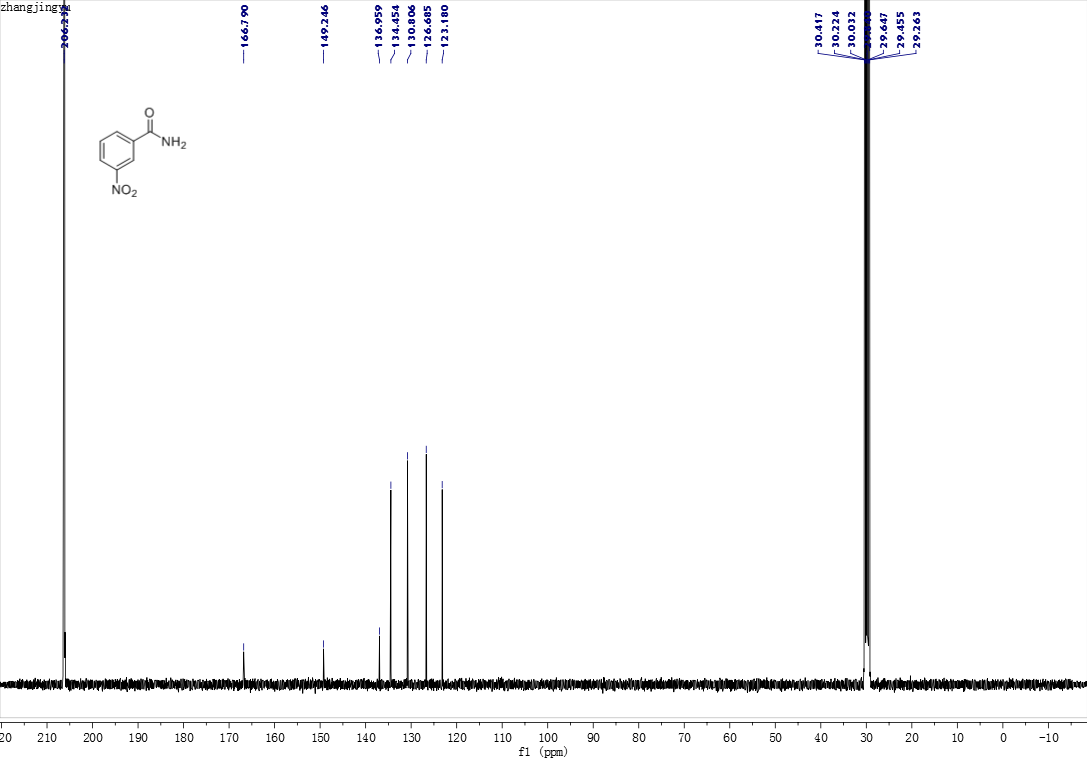


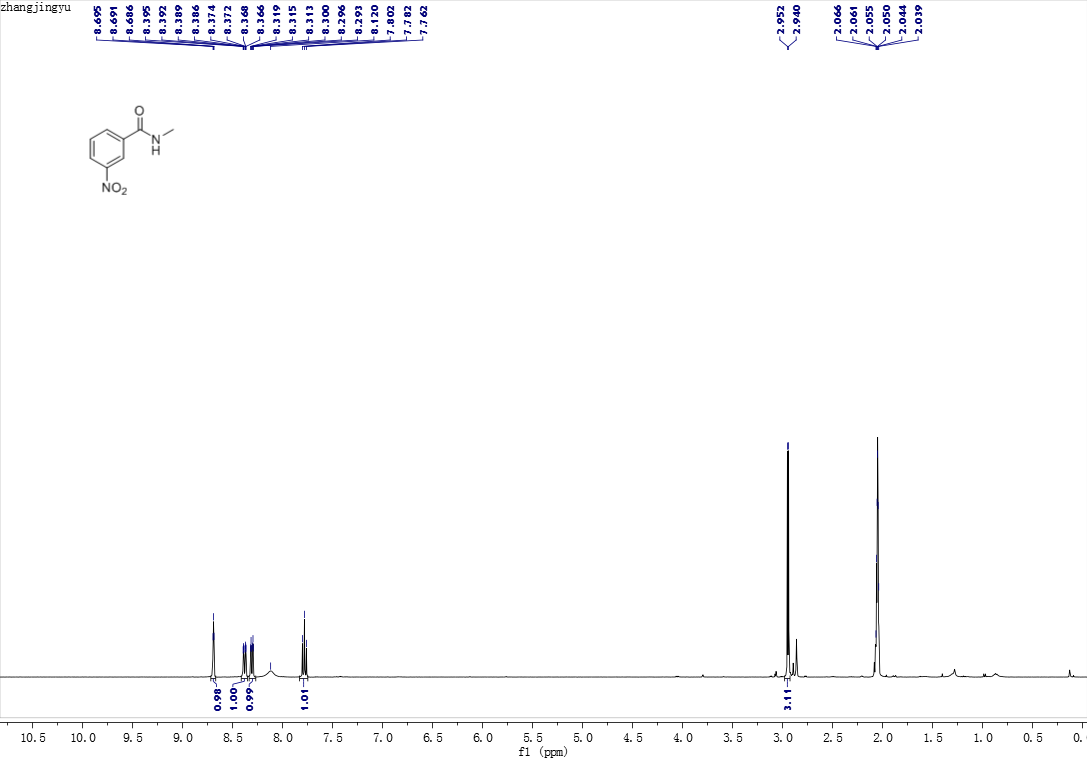


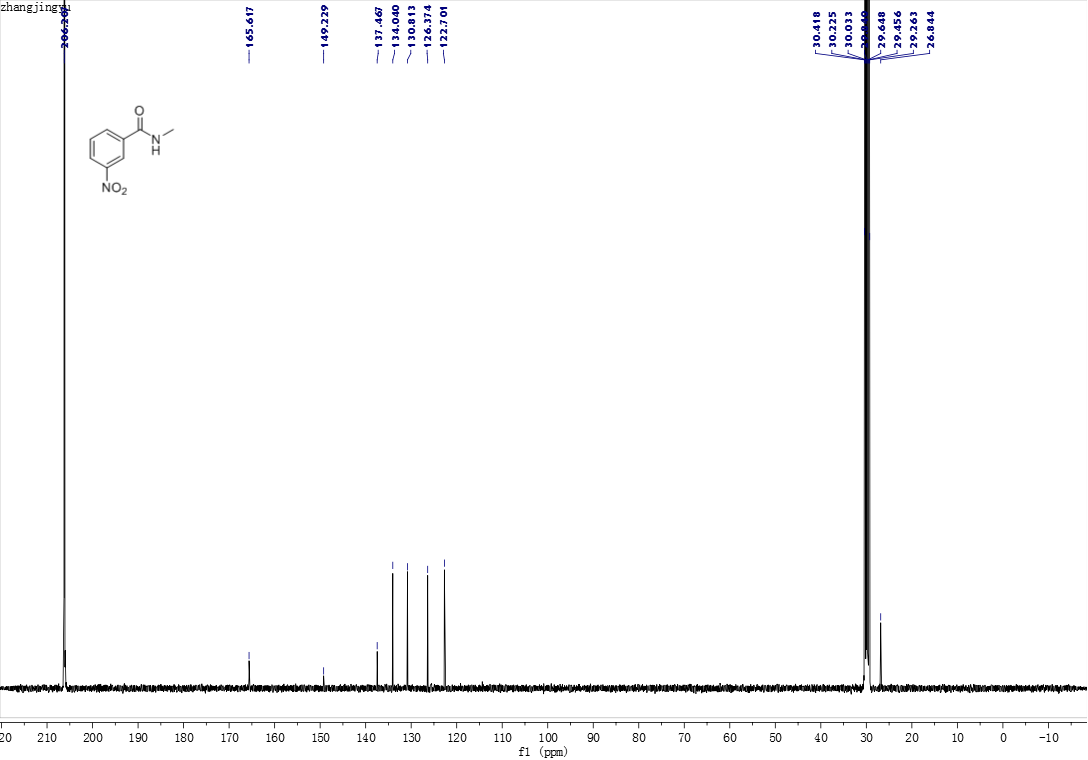


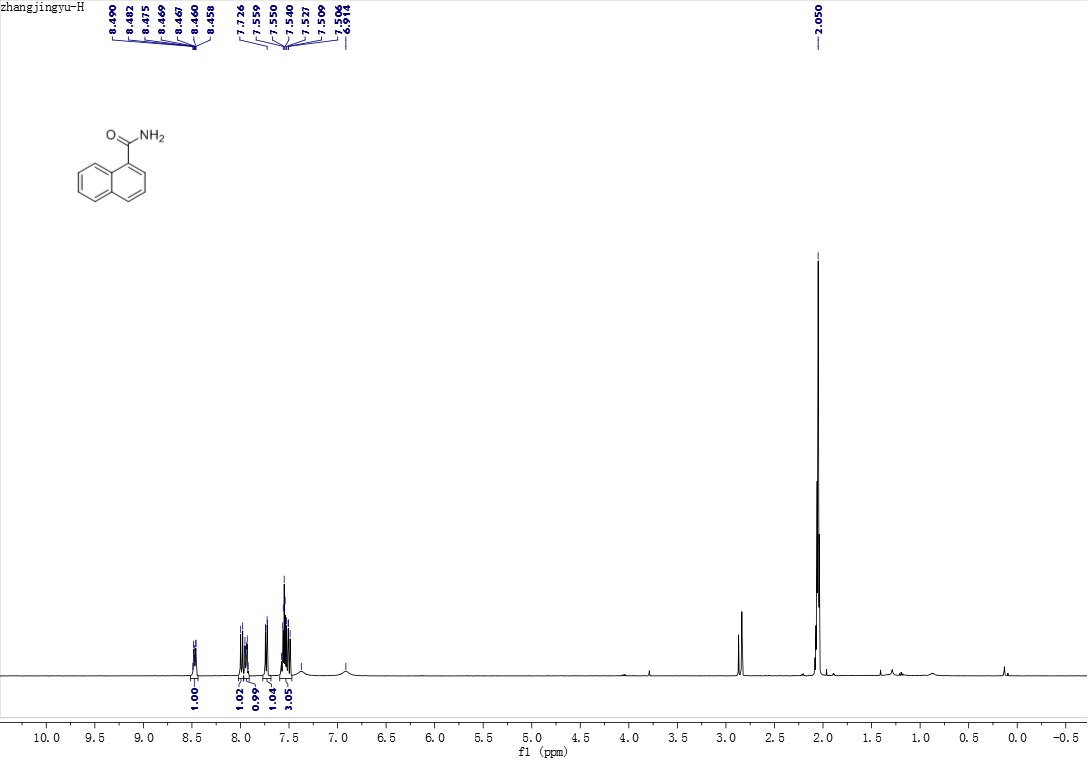


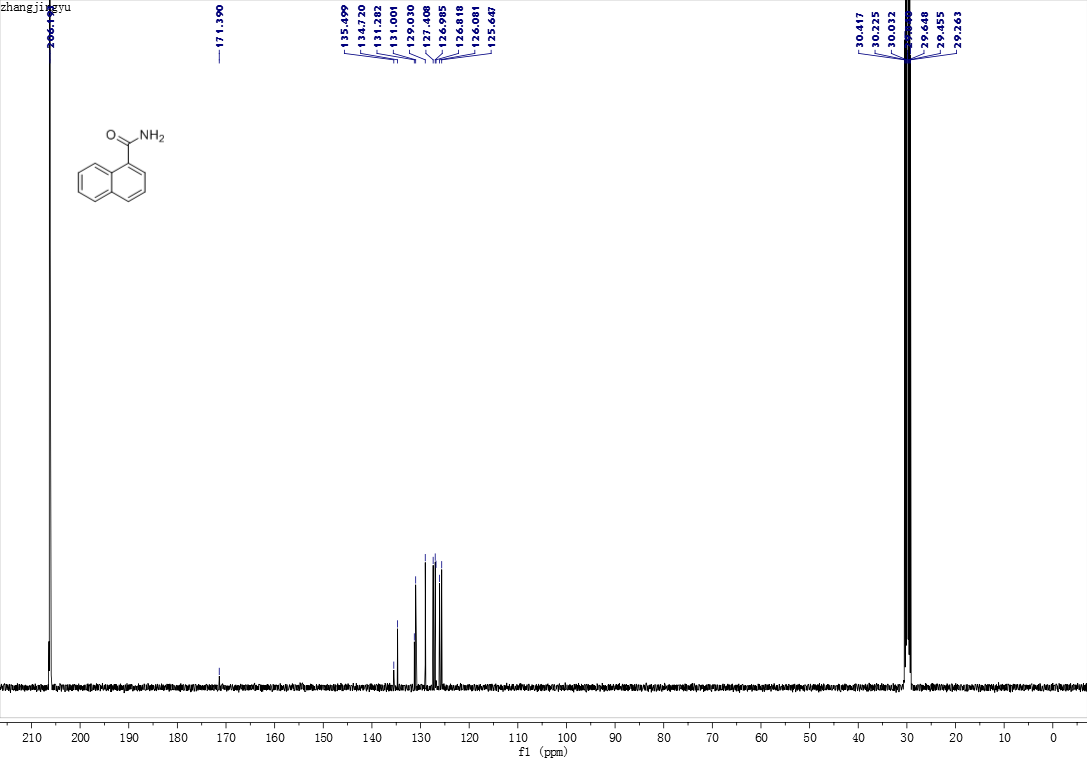


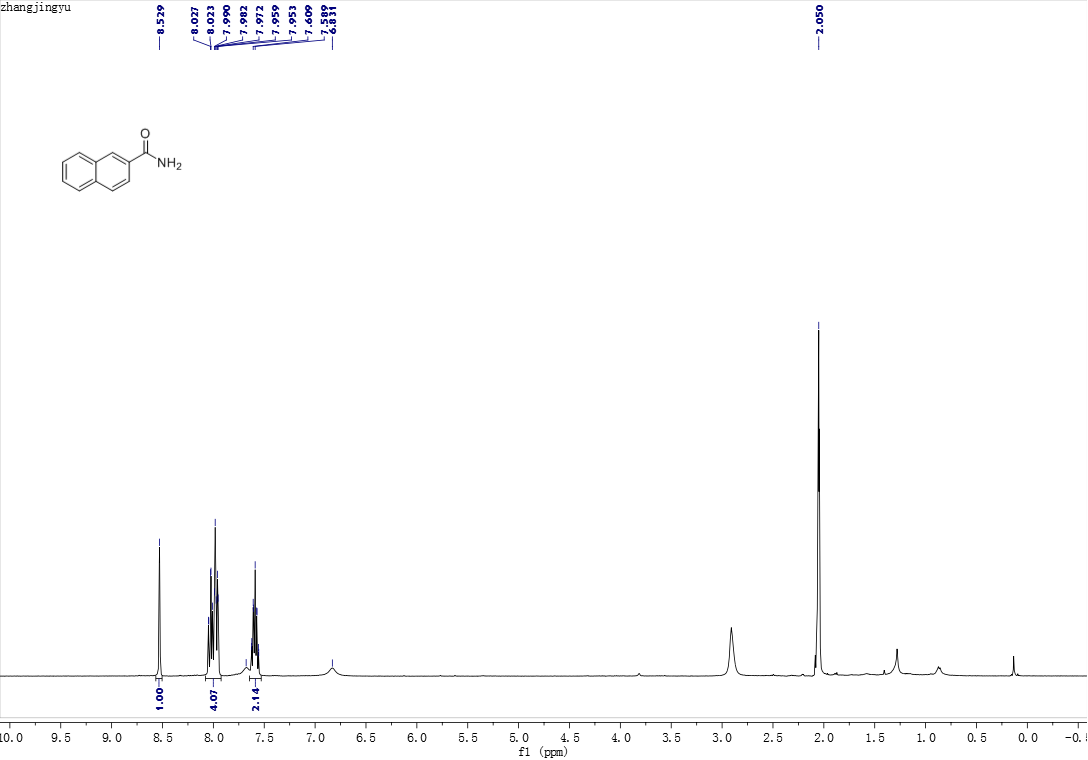


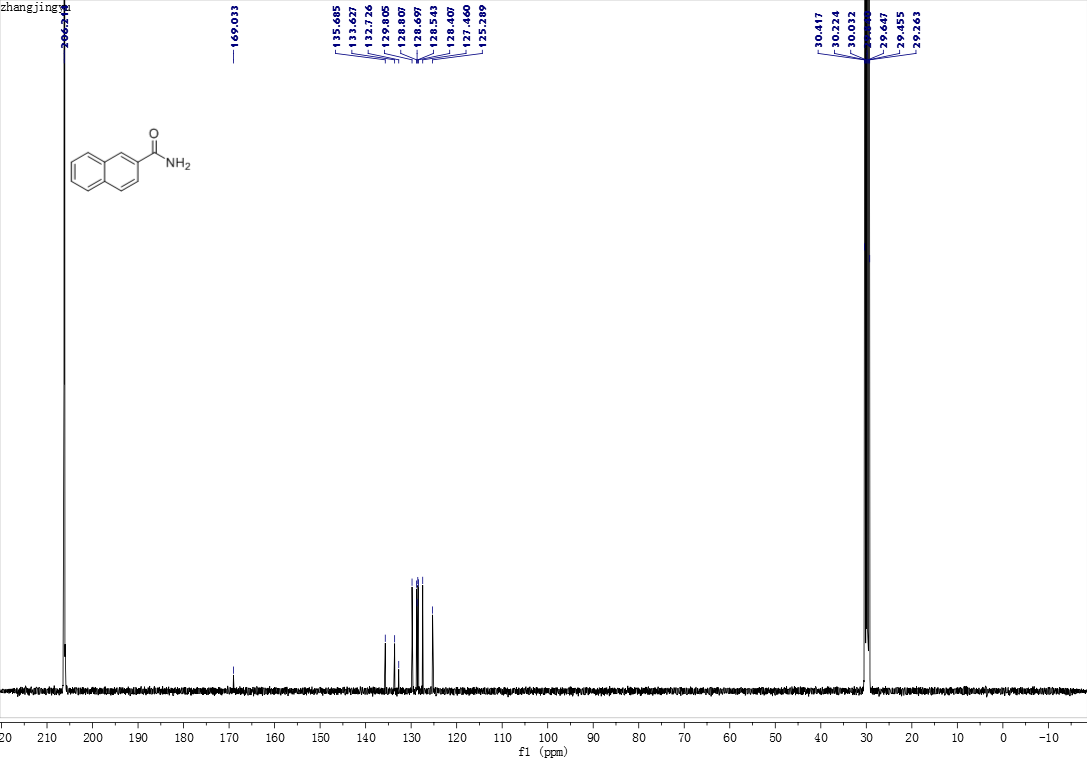


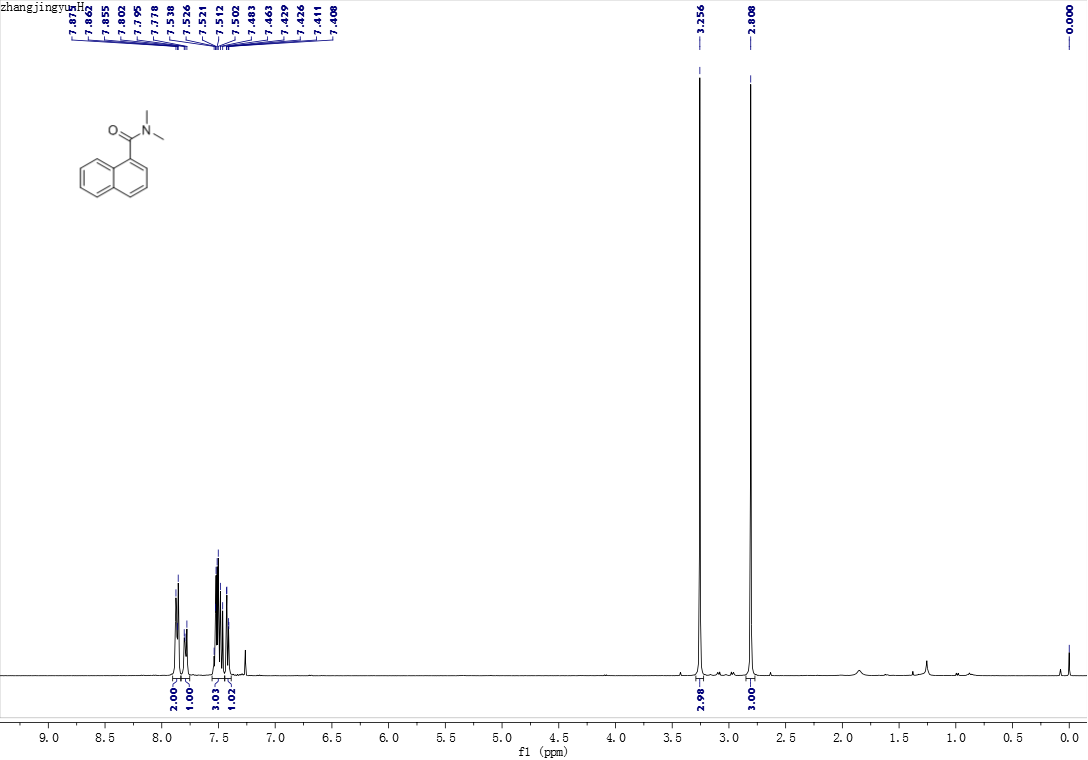


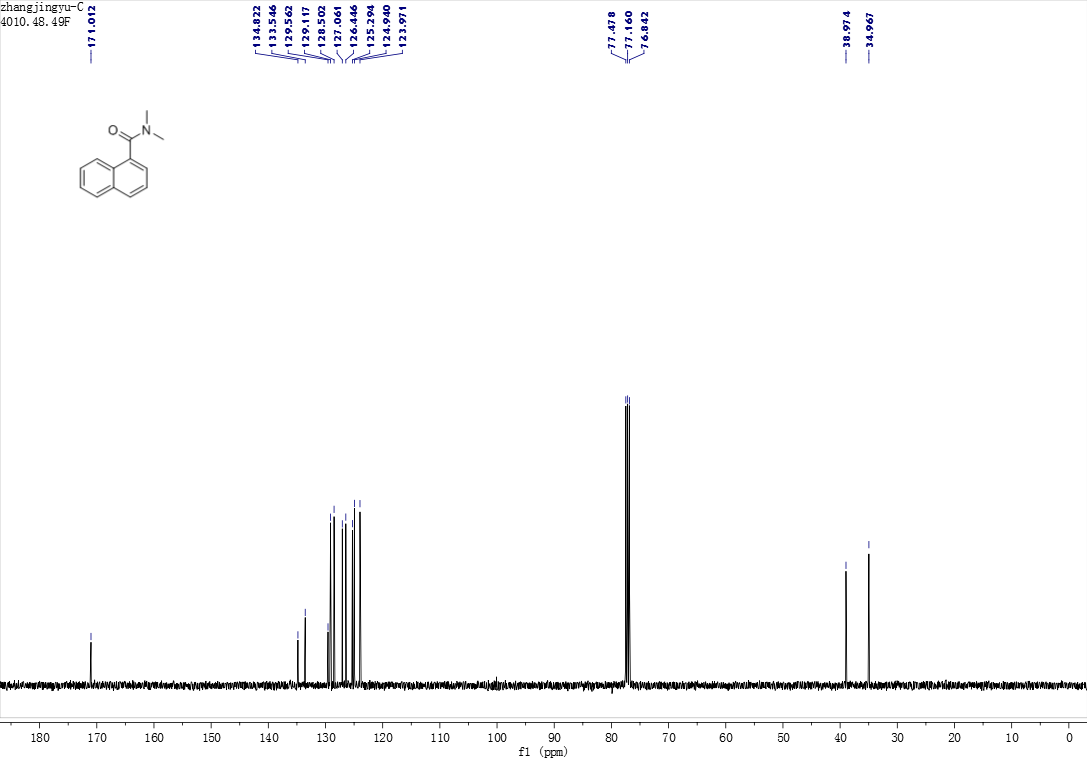


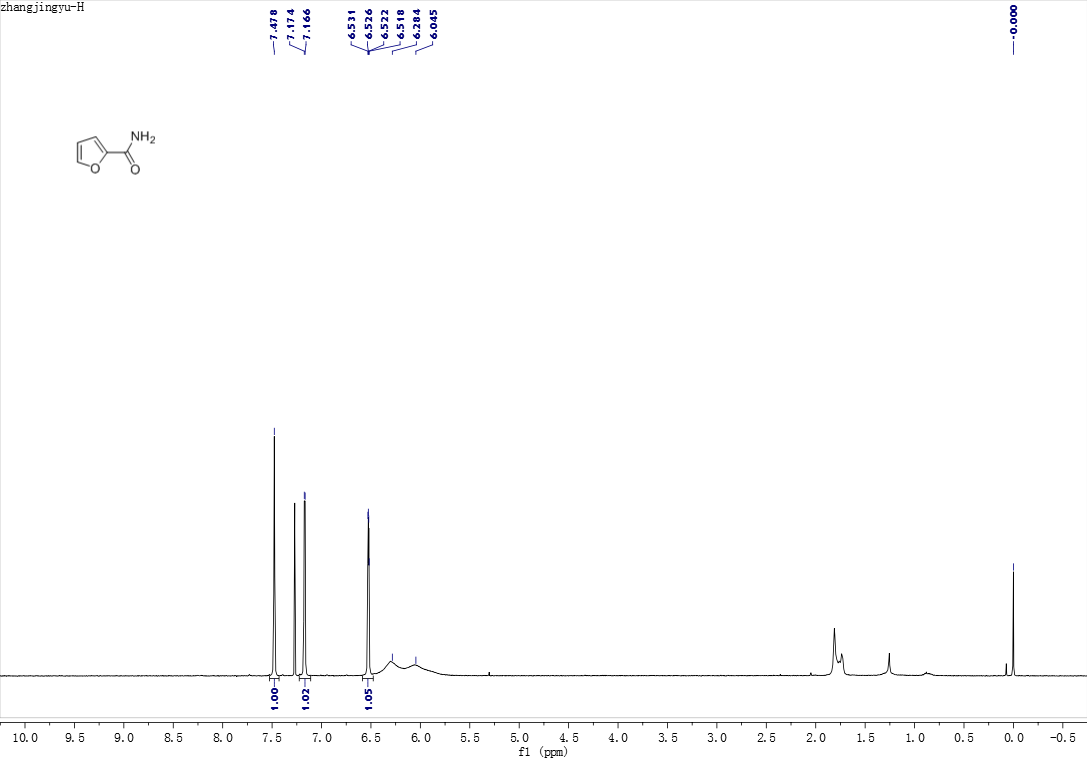


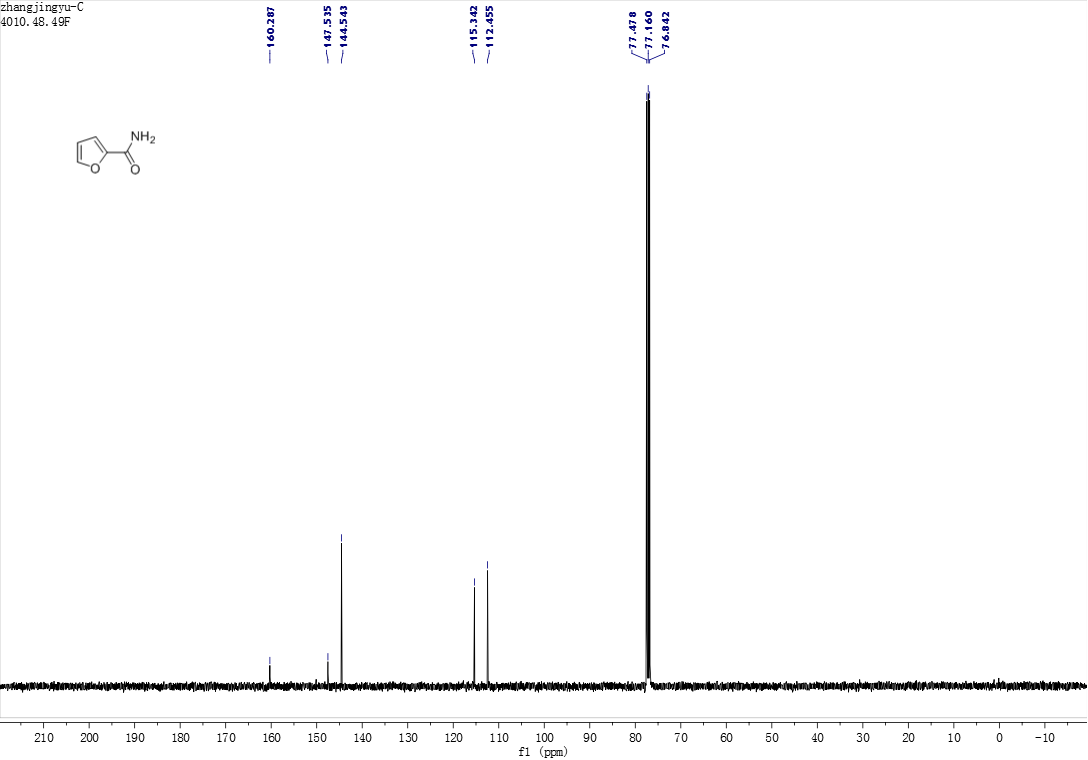


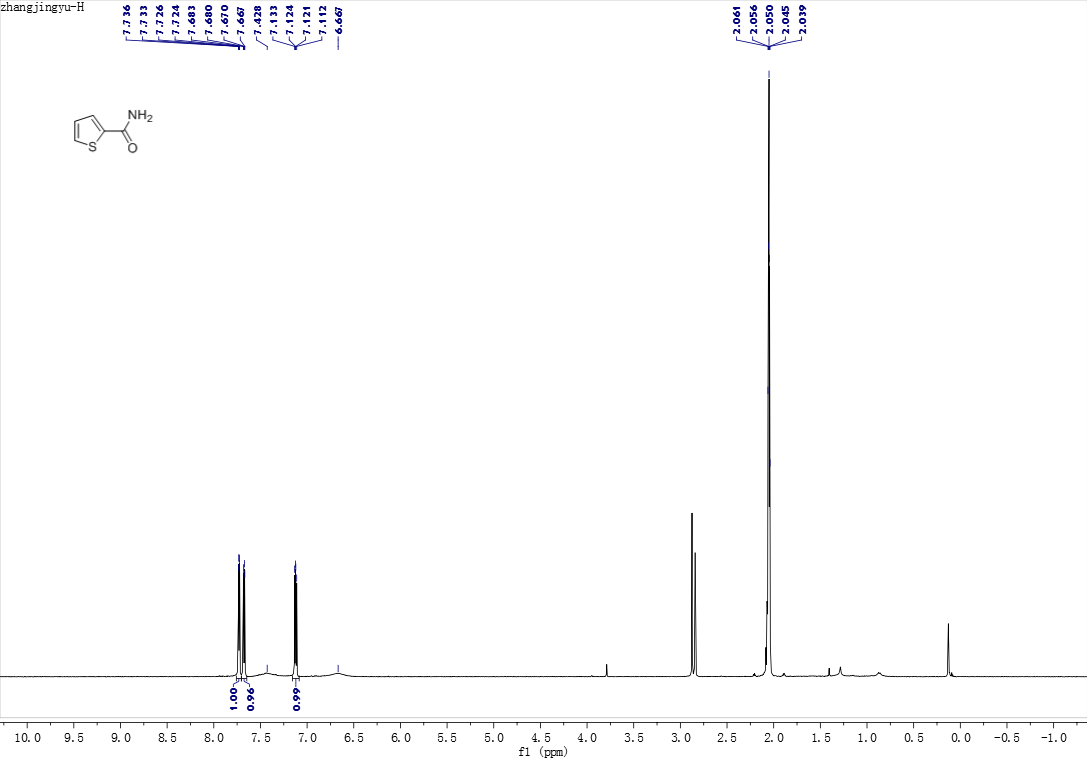


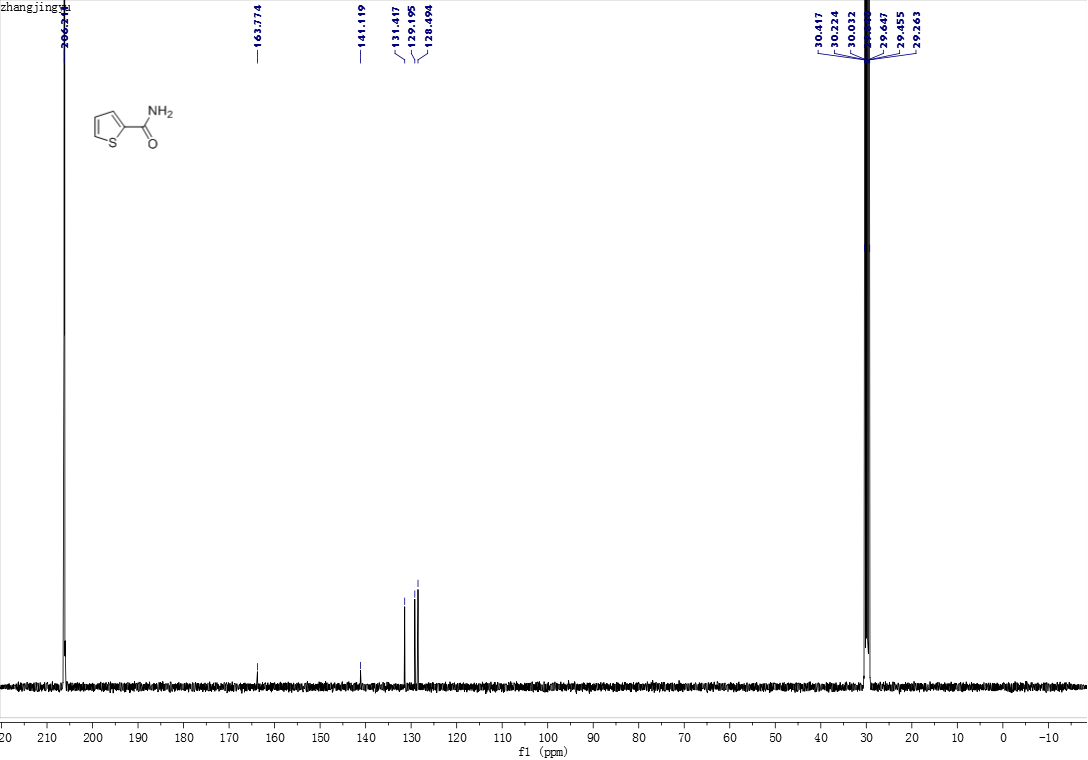


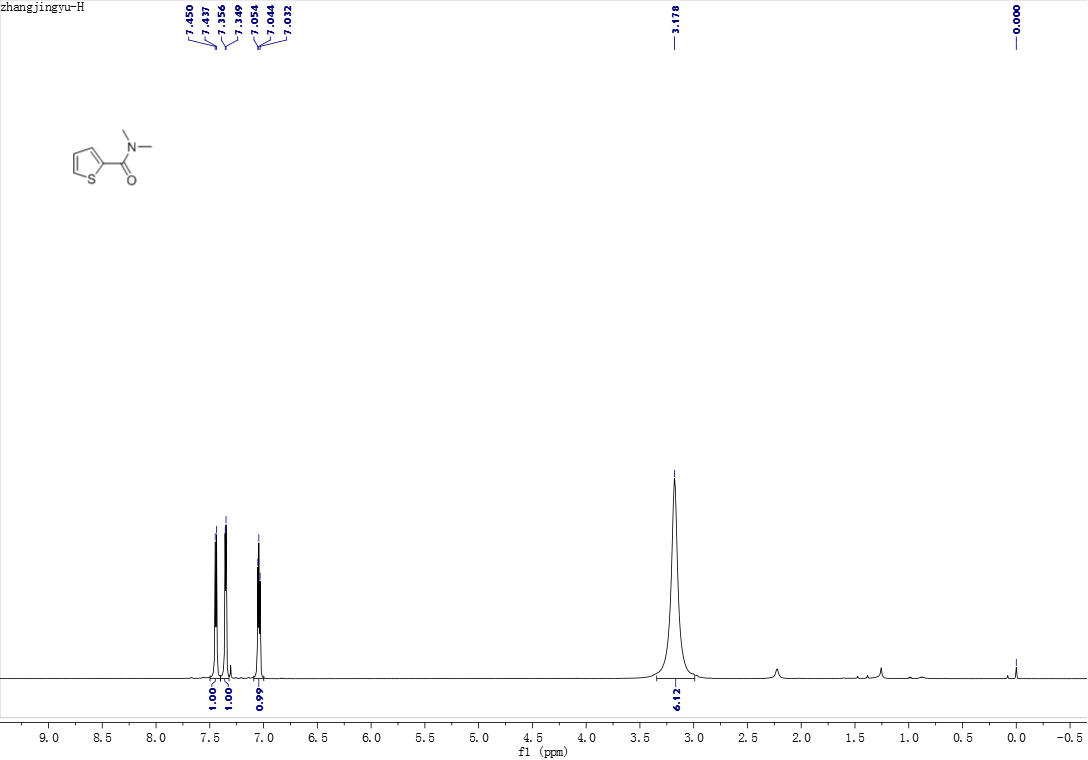


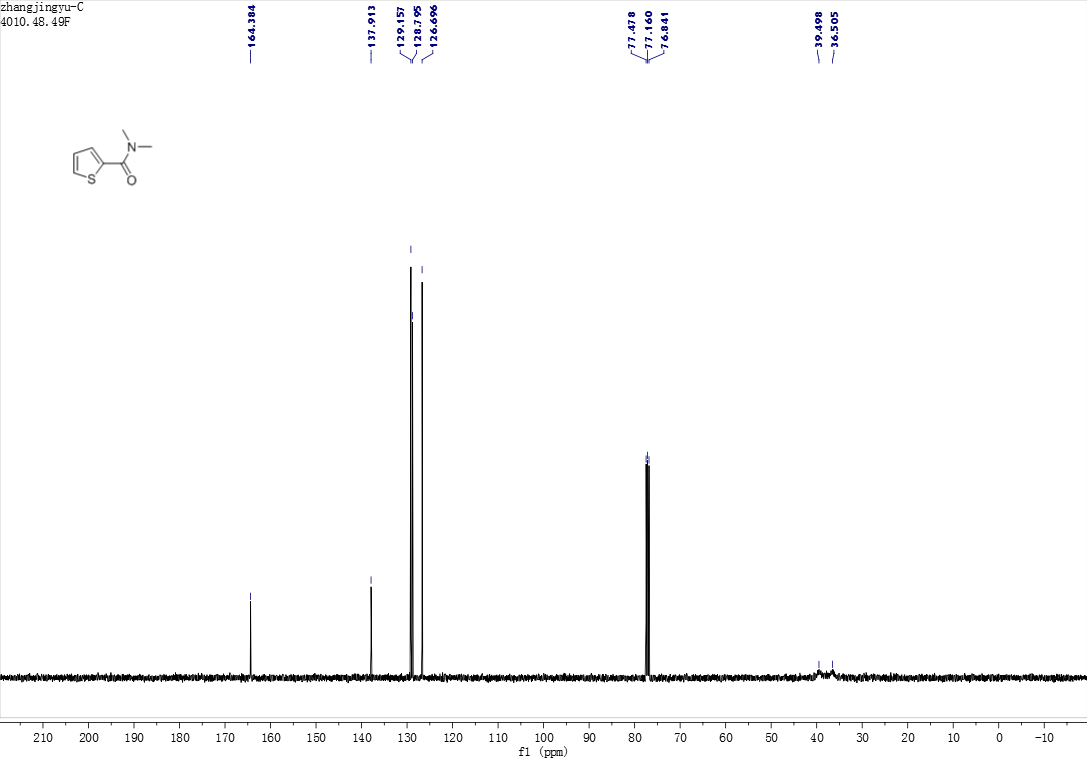


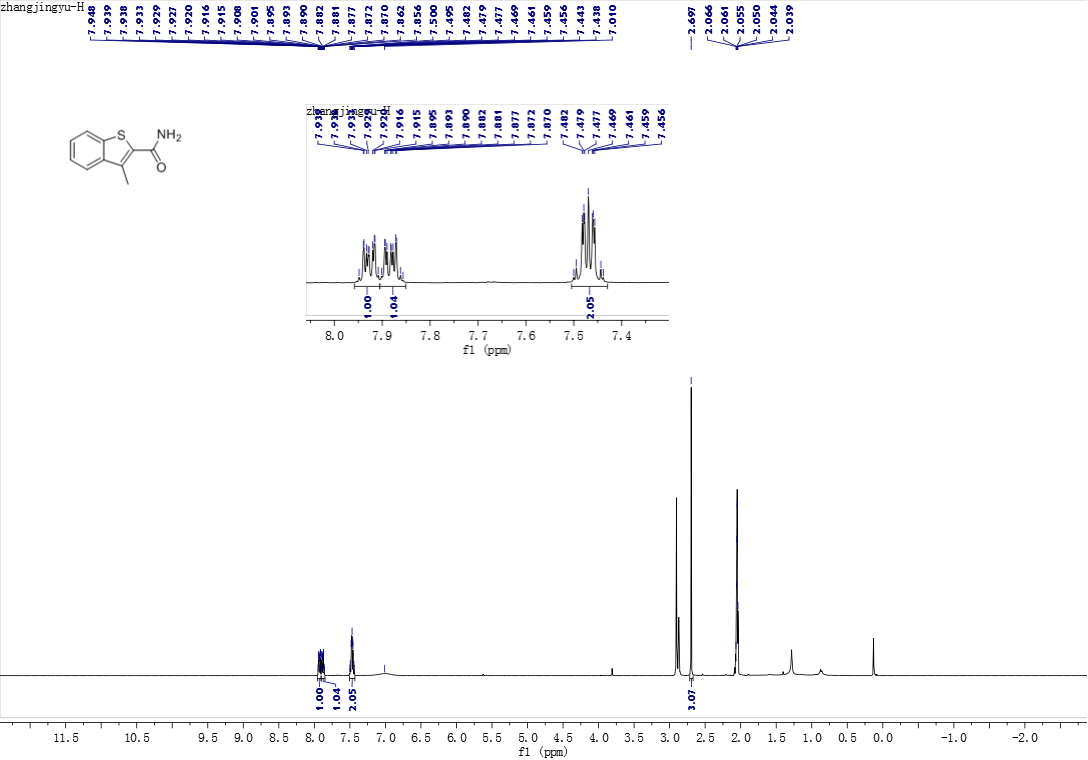


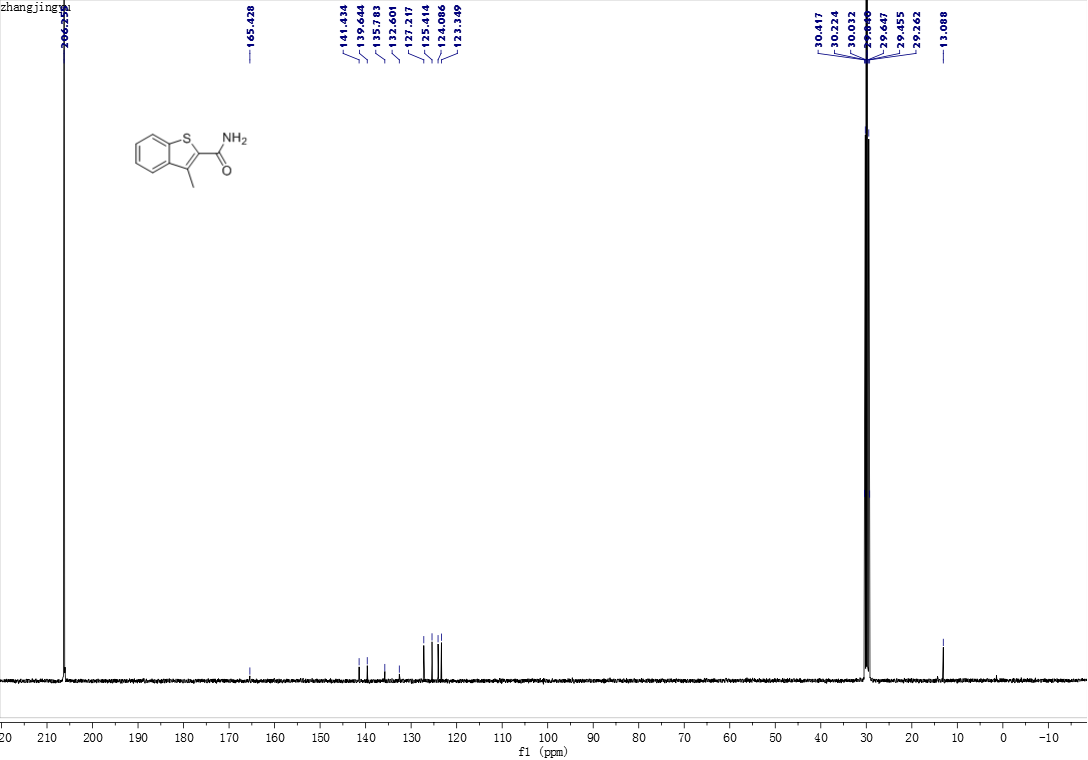


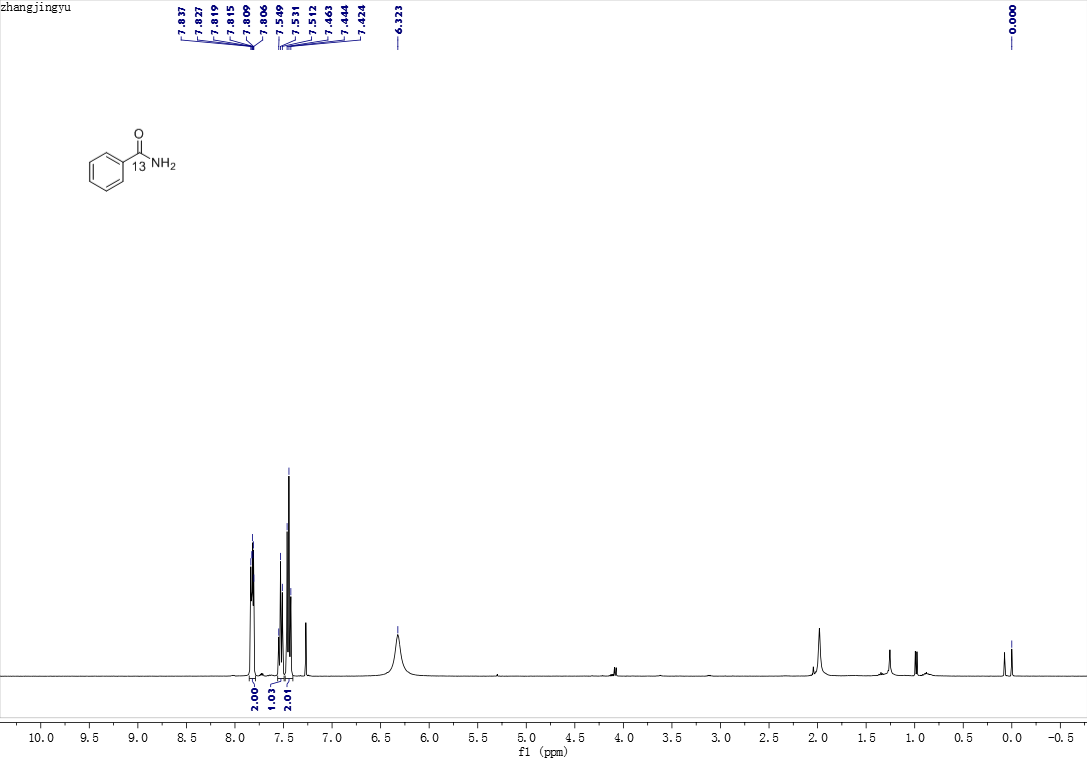


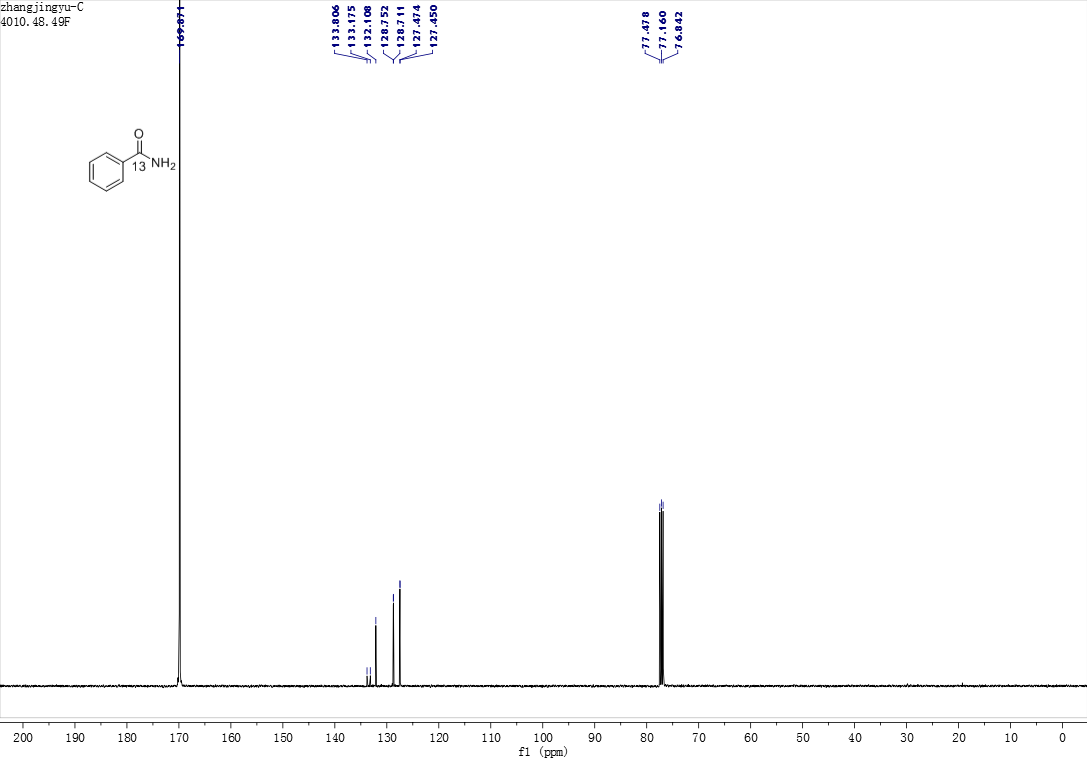

Supplement: Supplementary file 1 — Supplementary Information (traceless) [file 41598_2019_39240_MOESM1_ESM.docx]
